# Plastic Shrinkage Cracking of Full-Scale Concrete Slabs Under Field-Production Conditions: Observations on Mix Design, Environmental Exposure and Curing

**DOI:** 10.3390/ma19183899

**Published:** 2026-09-14

**Authors:** Gabriela Rutkowska, Barbara Francke, Mariusz Żółtowski, Eryk Ostrzyżek

**Affiliations:** Institute of Civil Engineering, Warsaw University of Life Sciences, Nowoursynowska 166 Street, 02-787 Warsaw, Poland; barbara_francke@sggw.edu.pl (B.F.); mariusz_zoltowski@sggw.edu.pl (M.Ż.); s218083@sggw.edu.pl (E.O.)

**Keywords:** plastic shrinkage cracking, full-scale concrete slabs, curing, evaporation rate, water-to-cement ratio, superplasticizer, hot-weather concreting, compressive strength

## Abstract

**Highlights:**

- Plastic shrinkage cracking was evaluated in 28 full-scale concrete slabs.
- Severe drying exposure increased the final crack count from 4 to 19.
- Solar heating increased the evaporation rate from 0.71 to 1.17 kg/m^2^/h.
- Severe W4 field exposure was associated with a final crack count of 19.
- Calculated potential evaporation rates were 0.71 kg/m^2^/h for initial W4 conditions and 0.22 kg/m^2^/h for W5.

**Abstract:**

PE sheeting produced the most consistent crack-free final observations under the investigated conditions. This study investigated the combined effects of mixture composition, water-reducing admixture system, environmental exposure, surface finishing, reinforcement configuration, and curing method on the early-age cracking and mechanical performance of concrete under full-scale production conditions. Seven concrete mixtures were investigated, and 28 full-scale reinforced concrete slabs were produced and monitored for plastic shrinkage cracking. Fresh-concrete workability, slump retention, 28-day compressive strength, environmental conditions, and surface evaporation rates were evaluated. The actual water-to-cement ratio ranged from 0.58 to 0.85, and increasing water demand following the reduction or elimination of water-reducing admixtures resulted in a decrease in 28-day compressive strength (150 mm cube specimens) from 41.4 to 24.6 MPa. Under the investigated full-scale field-production scenarios, visible plastic shrinkage cracking varied with the combined material, environmental, construction, and curing conditions. The unprotected W4-P1 slab, cast during severe hot and dry exposure, developed 19 visible cracks, whereas 4 cracks were recorded for W5-P1 under more moderate conditions. Because these slabs also differed in mixture composition and reinforcement configuration, this comparison is interpreted as an association rather than a single-factor environmental effect. The estimated evaporation rate was 0.71 kg/m^2^/h for the initial W4 conditions and 0.22 kg/m^2^/h for W5. Surface-finishing and reinforcement comparisons were based on individual slabs and are therefore reported only as case-specific observations. PE sheeting showed the most consistent performance under the investigated conditions: all nine PE-protected slabs were free of visible cracks at final inspection, while the two water-cured slabs developed zero or one crack. These findings show that visible plastic shrinkage cracking in full-scale production cannot be interpreted from mixture composition or w/c ratio alone and should be assessed in the context of the complete field-production scenario.

## 1. Introduction

Plastic shrinkage cracking is one of the earliest forms of damage that may develop in concrete elements. It occurs during the period between placement and setting, when concrete remains in a plastic or viscoplastic state and has not yet developed sufficient tensile strength to resist stresses generated by volumetric changes [1,2,3]. Elements with a large exposed surface relative to their volume, including slabs, industrial floors, pavements and bridge decks, are particularly susceptible to this phenomenon [2,3,4]. Although plastic shrinkage cracks originate at a very early stage, their consequences may persist throughout the service life of a structure by providing preferential pathways for the ingress of water and aggressive substances and thereby potentially accelerating deterioration processes [3,4,5]. Plastic shrinkage cracking is closely related to the moisture balance within the near-surface zone of fresh concrete. Following placement, settlement of solid particles and bleeding transport water toward the exposed surface. As long as the supply of bleed water compensates for moisture lost through evaporation, the surface remains saturated. When evaporation exceeds the rate at which water can be replenished, menisci form within the capillary pore system and negative capillary pressure develops [1,3,6]. The resulting contraction generates tensile stresses when deformation is restrained, and cracking may occur once these stresses exceed the very low tensile capacity of the young cementitious matrix [3,6,7]. Capillary-pressure measurements and modelling have therefore become important tools for understanding the onset of plastic shrinkage cracking. Recent research has demonstrated that critical capillary-pressure conditions can be identified and potentially used to predict or control crack formation [6].

The development of plastic shrinkage is strongly influenced by environmental exposure. High air and concrete temperatures, low relative humidity, solar heating and increased air velocity accelerate moisture loss from exposed concrete surfaces [8,9]. The simultaneous occurrence of these conditions can be particularly critical during hot-weather concreting, when the moisture balance of the surface may change rapidly. Alshammari et al. [8] demonstrated that increasingly severe environmental conditions resulted in a substantial increase in plastic shrinkage cracking and confirmed that temperature, relative humidity and wind velocity should be considered jointly when assessing cracking risk. Evaporation rate is therefore commonly used as an indicator of the potential for plastic shrinkage cracking. Classical approaches, including the model proposed by Uno [1], estimate evaporation from concrete and environmental temperatures, relative humidity and wind velocity. An evaporation rate of approximately 1.0 kg/m^2^/h has traditionally been associated with a high cracking risk. However, later investigations have shown that a single threshold cannot provide a universal criterion for modern concrete mixtures [3,8,10]. Cracking may occur at lower evaporation rates when bleeding is limited or when the available surface water is exhausted before sufficient stiffness and tensile resistance develop. Consequently, recent research has shifted from considering evaporation alone toward evaluating the time-dependent moisture balance between bleeding and evaporation. Kim and Kwak [10] developed an improved numerical approach in which both processes were incorporated into the prediction of surface drying and plastic shrinkage cracking. Their results indicate that moisture balance can provide a more physically representative indicator of cracking susceptibility than instantaneous evaporation rate alone and can potentially support on-site decisions regarding the timing of curing operations. Plastic shrinkage cracking is also strongly dependent on concrete mixture characteristics. Water-to-cement ratio, total water content, binder composition, supplementary cementitious materials and chemical admixtures influence bleeding, rheological behavior, setting, capillary-pressure development and early mechanical properties [11,12,13,14]. Recent research on high-performance cementitious mixtures confirms that rheological parameters and water-reducing admixtures can significantly affect plastic cracking resistance, highlighting the interaction between fresh-state behavior and early strength development [14]. Modern polycarboxylate-based superplasticizers make it possible to obtain the required workability while limiting mixing water. Their effectiveness in maintaining consistency is particularly important in ready-mixed concrete, where transport and placement time may result in progressive workability loss [15,16]. Compensating for such losses through uncontrolled addition of water can temporarily restore workability but simultaneously increases the water-to-cement ratio and may intensify bleeding and segregation while reducing the mechanical performance and durability of hardened concrete [12,15]. The influence of mixture design on plastic cracking therefore cannot be considered independently of environmental drying conditions. The complexity of these interactions has also been confirmed in full-scale experiments. Sayahi et al. [17] investigated the effects of cement type, water-to-cement ratio and chemical admixtures using concrete slabs measuring 3 m × 2 m. Their results demonstrated that plastic shrinkage cracking severity can be substantially modified by mixture composition and supported an assessment approach based on the relationship between evaporation and bleeding. Importantly, these experiments also illustrate the value of moving beyond small laboratory specimens when validating models intended to represent actual slab behavior. Construction operations performed after placement may constitute an additional source of variability. Surface finishing, consolidation and restraint by reinforcement can modify local settlement, bleeding and deformation of fresh concrete [3,18]. The state-of-the-art review by Kayondo et al. [3] identified settlement, bleeding, evaporation, capillary action and surface finishing as interacting mechanisms responsible for plastic cracking. Micro-computed tomography investigations have further shown that plastic shrinkage and settlement are three-dimensional phenomena and that voids may develop beneath reinforcing bars almost immediately after concrete placement [18].

The role of reinforcement in early-age cracking is therefore of particular interest. Roghani et al. [19], investigating reinforced concrete slabs under controlled evaporation conditions, showed that reinforcement configuration should be considered together with shrinkage and restraint when assessing early-age cracking behavior. Such findings emphasize that observations obtained from unreinforced laboratory specimens may not fully represent the mechanisms occurring in reinforced structural slabs. Among the practical measures available to mitigate plastic shrinkage, timely curing remains one of the most important. Its primary purpose at early age is to limit moisture loss from the exposed surface and maintain conditions favorable for cement hydration and microstructural development [20,21,22]. Common approaches include polyethylene sheets, wet coverings, water application and membrane-forming curing compounds. The effectiveness of curing compounds in reducing moisture loss has been demonstrated experimentally, although their performance depends on material properties, application rate and uniformity [20]. Alshammari et al. [21] directly investigated the effect of different curing procedures on plastic shrinkage cracking. Their results demonstrated substantial differences between curing strategies and showed that covering concrete with a plastic sheet prevented evaporation of bleed water and eliminated plastic shrinkage cracking under the investigated conditions. This finding highlights an important distinction between the intrinsic cracking susceptibility of a concrete mixture and the cracking actually observed when its exposed surface is adequately protected. The influence of curing extends beyond visible surface cracking. Bacharz et al. [22], investigating C30/37 concrete under different curing and environmental conditions, demonstrated relationships between shrinkage strain and microcracking processes monitored by acoustic emission. Their results indicate that early curing and environmental exposure can affect the subsequent development of internal damage, reinforcing the importance of controlling the conditions experienced by young concrete rather than considering curing solely as a means of preventing visible surface defects. Recent studies also demonstrate that the plastic shrinkage problem remains relevant as cement and concrete technologies evolve. Concrete containing high levels of clinker replacement may exhibit altered early hydration, bleeding and cracking behavior under rapid evaporation [23]. Research published in 2025 on calcined clay–limestone cement concretes showed greater plastic shrinkage and cracking under severe evaporation than conventional Portland cement concrete, illustrating how changes introduced to improve the environmental performance of binders may simultaneously modify their early-age moisture sensitivity. Various mitigation approaches have consequently been investigated, including fibers, modified binder systems and rheological control [5,14]. Alshammari et al. [5], for example, demonstrated that both manufactured and recycled steel fibers could reduce plastic shrinkage cracking under different environmental conditions. Such studies confirm that cracking results from interactions among material composition, moisture loss, restraint and early mechanical behavior rather than from a single isolated parameter. Despite substantial progress in understanding plastic shrinkage, a considerable proportion of experimental research continues to rely on controlled laboratory conditions designed to isolate individual variables. Such experiments are essential for identifying fundamental mechanisms and developing predictive models, but they cannot fully reproduce the simultaneous variability encountered during concrete production and construction. In practice, mixture composition, workability retention, air temperature, relative humidity, wind, surface temperature, finishing procedures, reinforcement configuration and curing effectiveness may change simultaneously.

Full-scale investigations have already demonstrated the value of assessing plastic shrinkage in larger elements [17], but the available literature remains comparatively limited with respect to experimental programmes that simultaneously combine controlled changes in concrete mixture composition with naturally varying environmental exposure, different finishing procedures and alternative curing strategies in a sufficiently large series of full-scale reinforced concrete slabs. This distinction is important because the factor governing cracking under one environmental condition may cease to be dominant when evaporation intensity or surface protection changes.

Therefore, the objective of the present study was to document and evaluate the behavior of full-scale concrete slabs produced under realistic ready-mixed concrete plant conditions, with particular attention to mixture composition, environmental exposure, surface-finishing practice, reinforcement configuration, and curing method. The experimental program comprised seven concrete mixtures designed for strength class C30/37 and a total of 28 reinforced slabs measuring 3.0 m × 1.5 m × 0.15 m. Fresh-concrete behavior, environmental conditions, visible crack occurrence, and 28-day cube compressive strength were evaluated within the same field-production program. The principal value of the study lies in integrating material and construction observations at full scale under naturally varying production conditions. Because the factors were not varied independently in a factorial design, the study does not quantify causal interactions or rank the independent effects of individual variables. Instead, it provides comparative field-case observations and tests the practical hypothesis that unfavorable drying scenarios may be associated with increased visible cracking, while timely surface protection may be associated with reduced final crack occurrence.

## 2. Materials and Methods

### 2.1. Materials

All concrete mixtures were prepared using CEM II/B-M (S-V) 42.5 R Portland-composite cement (Cemex, Chełm, Poland), containing granulated blast-furnace slag (S) and siliceous fly ash (V). The cement had a specific density of 2.94 g/cm^3^ and a specific surface area of 4835 cm^2^/g. The initial setting time was 218 min, while the mean compressive strength reached 25.1 MPa after 2 days and 56.0 MPa after 28 days. The SO_3_ content was 2.88%, complying with the requirements of EN 197-1:2012 [24]. Siliceous fly ash originating from the Kozienice Power Plant (Veolia EKOZEC, Kozienice, Poland) was used as an additional mineral component and complied with the requirements of EN 450-1:2012 [25]. The declared particle density was 2100 kg/m^3^, while the fineness, expressed as the residue on a 0.045 mm sieve, was 30%. The aggregate skeleton consisted of three fractions: natural sand 0–2 mm obtained from the Janowiec quarry (Janowiec, Poland), and crushed dolomite aggregates with particle-size fractions of 2–8 and 8–16 mm originating from the Łagów V quarry (Łagów, Poland). The natural sand corresponded to grading category GF85 in accordance with EN 12620+A1:2010 [26]. Both coarse aggregate fractions had a particle density of 2.67 Mg/m^3^ and water absorption of 1%. Resistance to fragmentation corresponded to category LA25, whereas the flakiness and shape indices complied with categories FI20 and SI20, respectively. Three chemical admixtures complying with EN 934-2+A1:2012 [27] were used to modify the rheological properties of the concrete mixtures. CHRYSO Plast 229 was a plasticizer based on modified lignosulfonates, whereas CHRYSO Delta 131 and MC-TechniFlow 25 were polycarboxylate ether (PCE)-based superplasticizers. In addition, CHRYSO Cure HPP, a paraffin-dispersion-based membrane-forming curing compound, was used for selected slabs (CHRYSO, Warsaw, Poland).

### 2.2. Concrete Mix Design

The reference mixture was a ready-mixed concrete designed for strength class C30/37. A target consistency corresponding to class S3 was adopted for all concrete variants. The principal technological assumption was to maintain a comparable level of workability while systematically modifying the type and dosage of water-reducing admixtures. This approach enabled the effects of reducing or eliminating chemical admixtures on water demand, the actual water-to-cement ratio, workability retention, compressive strength, and susceptibility to plastic shrinkage cracking to be evaluated. Seven concrete mixtures, designated W1–W7, were investigated. W1 was the reference mixture containing CHRYSO Plast 229 plasticizer and CHRYSO Delta 131 superplasticizer. In W2, the superplasticizer was omitted and the plasticizer dosage was increased. In W3 and W4, the plasticizer dosage was reduced to approximately half that used in W2. During production of W3, an unintended ingress of water from washing of the mixer hopper occurred. This technological incident was documented and the mixture was retained in the experimental program as a separate field-production scenario. W4 was subsequently produced using the same nominal admixture strategy but without this unintended water addition. W5 was produced without water-reducing admixtures. In W6, the cement content was increased and a plasticizer was used, whereas W7 incorporated the alternative PCE-based superplasticizer MC-TechniFlow 25 (MC-Bauchemie, Środa Wlkp., Poland). The actual quantities of constituents dosed during concrete production are presented in Table 1. Actual production data were used rather than nominal mixture proportions because they correspond directly to the concrete used for preparation of the full-scale slabs.

Aggregate moisture was accounted for during concrete production. Depending on the testing day, the moisture content ranged from 3.0 to 5.0% for sand and from 1.0 to 1.5% for both coarse aggregate fractions. The total water contents reported in Table 1 include both the water added during batching and the water introduced with the moist aggregates. Consequently, the actual w/c ratio ranged from 0.58 for W1 to 0.85 for W5, despite the intention to maintain a comparable consistency among the investigated mixtures.

### 2.3. Experimental Program

**Figure 1:**
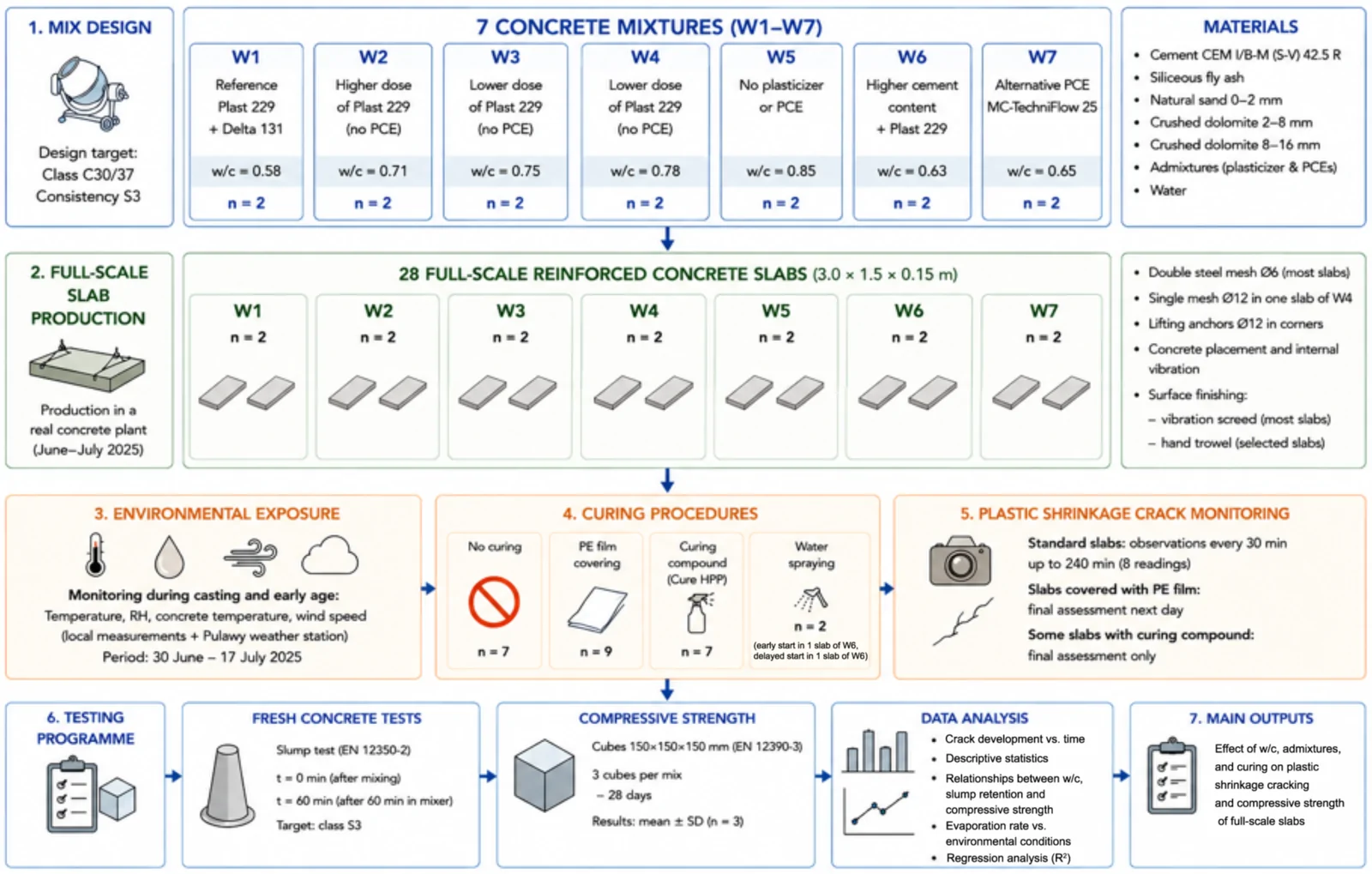
Schematic representation of the experimental program including seven concrete mixtures, 28 full-scale reinforced concrete slabs, environmental exposure, curing procedures, and fresh and hardened concrete testing.

The experimental program was designed as a full-scale field-production investigation of concrete mixture composition, actual environmental exposure, surface-finishing procedures, reinforcement configuration, and curing practice in relation to visible plastic shrinkage cracking. The tests were carried out under actual production conditions at a ready-mixed concrete plant. The program comprised seven concrete mixtures (W1–W7) and a total of 28 full-scale reinforced concrete slabs. The number of slabs assigned to individual mixtures was intentionally non-uniform because W6 and W7 were used for a more extensive assessment of curing procedures. Fresh-concrete properties and compressive strength were evaluated in parallel, while environmental conditions and crack development were monitored during the full-scale tests. The program was not a factorial experiment: several material, environmental, and construction variables changed simultaneously, and selected comparisons were represented by only one slab per condition. Accordingly, these comparisons are treated as field case observations rather than statistically independent tests of individual factors. The overall experimental workflow is presented in Figure 1. Generative artificial intelligence (GenAI) tools were used solely to assist in the graphical preparation of Figure 1. All scientific content and data were provided, reviewed, and verified by the authors, who take full responsibility for the accuracy of the final figures.

The distribution of the slabs among the individual mixture variants and the principal additional experimental variables are summarized in Table 2. W2–W5 comprised two slabs each, whereas W6 included ten slabs and W7 eight slabs.

### 2.4. Production of Full-Scale Concrete Slabs

Each slab had dimensions of 3.0 m × 1.5 m × 0.15 m. The slabs were cast in prepared molds located in a designated experimental area within the concrete plant premises. Standard slabs were reinforced with a double steel mesh made of Ø6 mm bars supported on spacers. One slab from mixture W4 was provided with a single mesh made of Ø12 mm bars to enable an additional assessment of the influence of reinforcement configuration on early-age cracking. The corresponding W4 slab contained the standard double Ø6 mm reinforcement. Four lifting anchors made of Ø12 mm ribbed bars were installed at the corners of each element. The concrete mixture was placed directly into the molds and consolidated using an ENAR M 5 AFP ((ENARCO S.A., Zaragoza, Spain) internal vibrator equipped with a 50 mm diameter poker. A KREBER (GmbH, Erkrath, Germany) vibrating screed with a working width of 2000 mm was used for surface consolidation and levelling of most slabs. Selected elements were manually finished using a steel hand trowel, providing a case-specific observational comparison of surface-finishing procedures; because only one slab represented each finishing condition, no statistical inference regarding finishing technique was possible.

**Figure 2:**
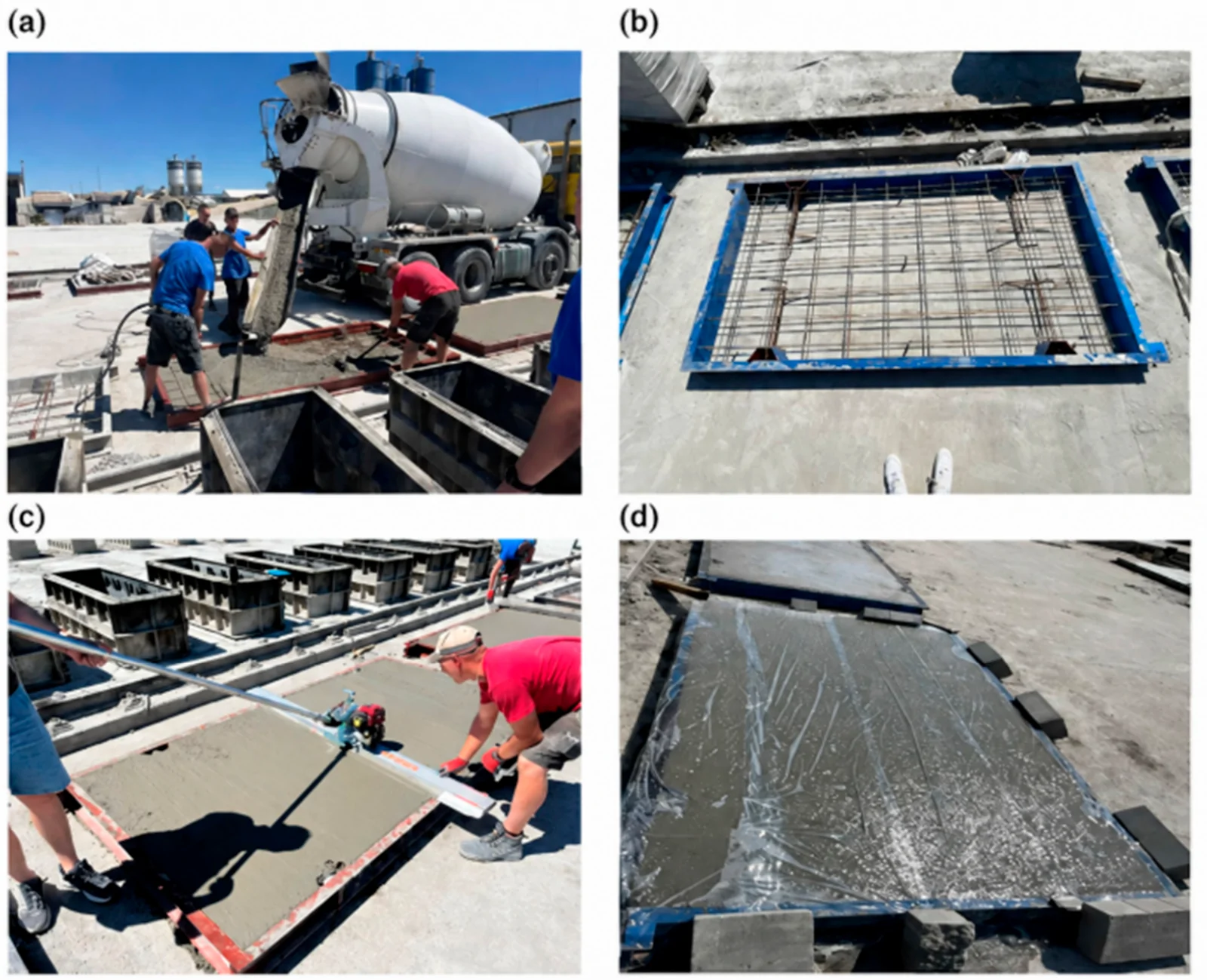
Main stages of the full-scale experimental program: (**a**) concrete placement and consolidation; (**b**) reinforcement arrangement of the test slab; (**c**) surface finishing using a vibrating screed; (**d**) early-age curing using PE sheeting.

The main stages of slab preparation and testing are illustrated in Figure 2.

### 2.5. Aggregate Grading Verification

**Figure 3:**
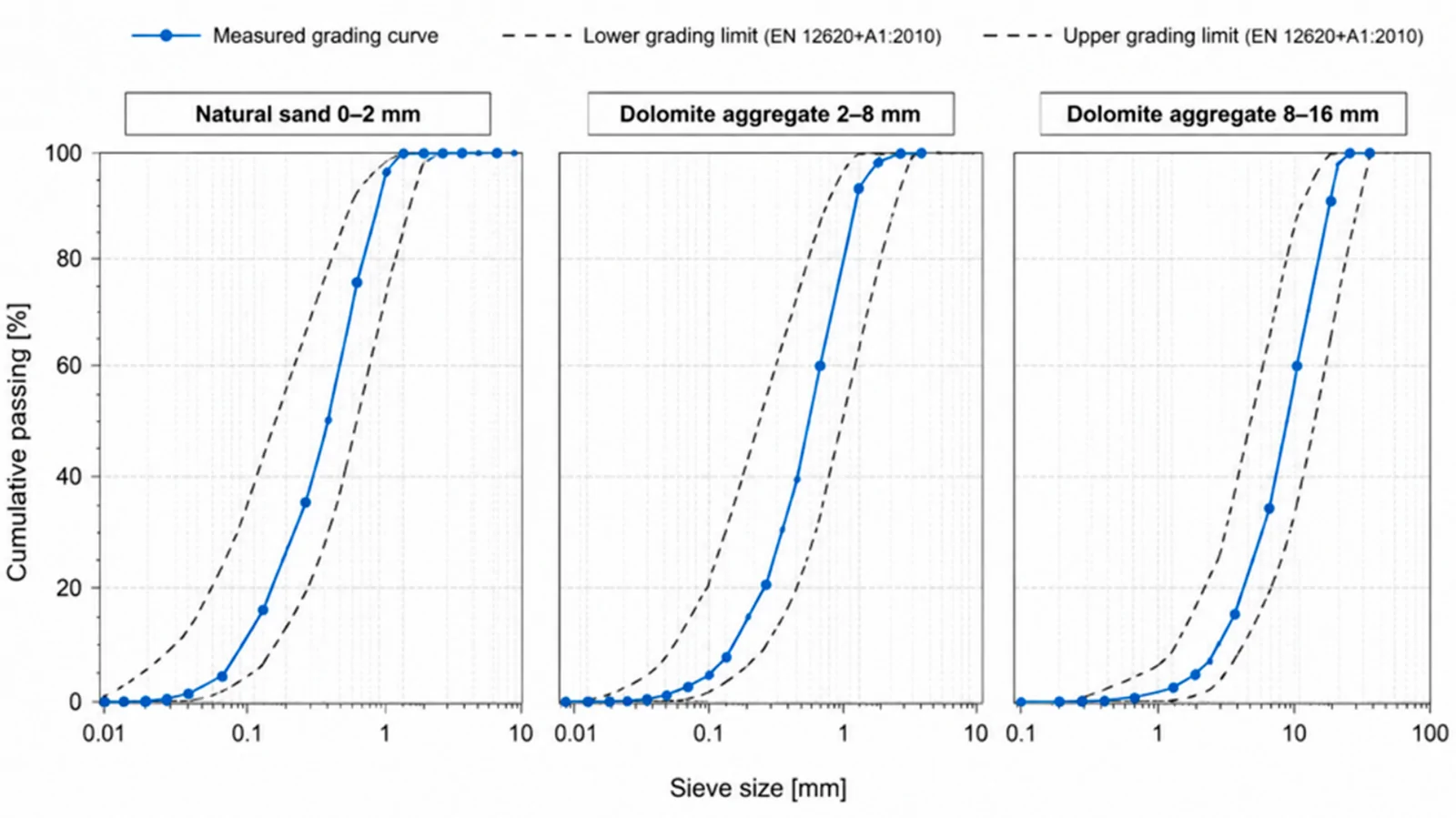
Particle-size distribution curves of the aggregates used in the study together with the corresponding grading limits.

The particle-size distributions of the aggregates were determined in accordance with EN 933-1:2012 [28] using the wet-sieving method with a standardized set of square-mesh sieves. The resulting grading curves were compared with the corresponding grading limits specified in EN 12620+A1:2010 [26]. The purpose of this verification was particularly important in the present investigation because aggregate grading and excessive fines may influence water demand and fresh-concrete rheology. Confirming the consistency of aggregate grading therefore reduced the possibility that differences in water demand among the mixtures resulted from uncontrolled aggregate characteristics rather than from the investigated admixture modifications. For the 0–2 mm natural sand, the grading curve remained within the relevant grading envelope and the content of particles smaller than 0.063 mm was 0.7%. The grading curves of the 2–8 and 8–16 mm dolomite fractions also generally followed the corresponding grading ranges, with only minor deviations. The particle-size distribution curves are presented in Figure 3.

### 2.6. Environmental Conditions

The full-scale slab tests were conducted during the summer period from 30 June to 17 July 2025. During casting and the early-age observation period, air temperature, relative humidity, concrete mixture temperature, and wind velocity were monitored locally. Local measurements were supplemented by meteorological data obtained from a certified weather station located in Puławy, Poland. The exposure conditions varied considerably among the individual test series. Air temperature ranged from approximately 19.8 °C for W7 to 35.2 °C for W4, relative humidity ranged from 28 to 71%, and wind velocity ranged from 0.3 to 5.1 m/s (Table 3).

These ranges characterize the field conditions associated with each production series; because the mixtures were produced on different days and the environmental variables were not independently controlled, weather-related comparisons are interpreted as scenario-based associations rather than isolated environmental effects. The available experimental record supports series-level environmental ranges rather than a fully time-resolved casting chronology for every individual slab, which is acknowledged as a limitation of the field-production design.

The wide range of exposure conditions enabled the behavior of the concrete mixtures to be assessed under both moderate environmental conditions and severe drying conditions characteristic of hot summer weather.

### 2.7. Curing Procedures

Four surface-protection conditions were investigated: no curing, covering with polyethylene (PE) sheeting, application of the CHRYSO Cure HPP membrane-forming compound, and water curing by surface spraying. PE sheeting was applied as soon as the surface condition allowed covering without mechanically damaging the freshly finished concrete. The edges of the sheets were secured to minimize air exchange and prevent displacement by wind. CHRYSO Cure HPP was spray-applied after disappearance of the visible surface-water sheen. The application rate was maintained within the manufacturer’s recommended range of 100–200 mL/m^2^. For water curing, water with a temperature as close as practically possible to that of the concrete surface was used to minimize sudden thermal gradients. The influence of curing initiation time was additionally investigated within W6. On one slab, water spraying was initiated as soon as the surface had developed sufficient resistance to avoid damage, whereas on another slab the start of water curing was deliberately delayed by 90 min. Because only one slab represented each curing-initiation condition, this comparison was treated solely as a case-specific field observation and was not subjected to inferential statistical analysis. Within W6, slabs W6-P1 and W6-P4 were subjected to water curing, while the remaining selected slabs were protected either with PE sheeting or with the membrane-forming compound. W7 slabs were protected with PE sheeting. The extended number of specimens within W6 and W7 was therefore used primarily to assess the practical effectiveness of different surface-protection strategies.

### 2.8. Fresh Concrete Testing

The consistency of all concrete mixtures was determined using the slump test in accordance with EN 12350-2:2019 [29]. A target consistency class S3 was adopted. Two measurements were performed for each concrete mixture. The first measurement was carried out immediately after production (t = 0 min). The second measurement was performed after 60 min (t = 60 min). Between the two measurements, the concrete remained in the mixer drum, which was continuously rotated at minimum rotational speed. This procedure was intended to simulate the conditions associated with prolonged transport of ready-mixed concrete and enabled the workability retention of the different admixture systems to be compared. Slump loss after 60 min was additionally expressed as the absolute decrease in slump and as the percentage reduction relative to the initial value.

### 2.9. Compressive Strength Testing

Concrete compressive strength was determined in accordance with EN 12390-3:2019-07 [30] using cubic specimens measuring 150 mm × 150 mm× 150 mm. The specimens were prepared in molds complying with EN 12390-1:2021-12 [31] and cured in accordance with EN 12390-2:2019-07 [32]. For each concrete variant, three cubic specimens were tested after 28 days. The specimens were mechanically consolidated on a vibrating table, demolded after initial hardening, and subsequently stored immersed in water at approximately 18 °C until testing. The 28-day compressive strength was expressed as the arithmetic mean ± standard deviation (*n* = 3).

### 2.10. Assessment of Plastic Shrinkage Cracking

Plastic shrinkage cracking was assessed by systematic visual inspection of the upper surface of the full-scale slabs. For the purposes of the present experimental programme, a crack was defined as a macroscopic discontinuity visible to the naked eye with a minimum length of 5 cm. The procedure represented an adaptation of general recommendations concerning plastic cracking to full-scale field elements [33]. For standard slabs without an impermeable covering, observations commenced immediately after completion of surface finishing and continued for the first 4 h. Inspections were carried out at 30-min intervals, i.e., after 30, 60, 90, 120, 150, 180, 210, and 240 min. The time of appearance of the first visible crack and the subsequent increase in crack number were recorded. The principal quantitative parameter was therefore visible crack count, rather than crack width, total crack length, crack area, or spatial crack distribution. Consequently, the term “cracking severity” is not used for the present measurements, because equal crack counts may represent substantially different crack geometries and engineering significance. For slabs protected with PE sheeting, repeated observations at 30-min intervals were not performed because removal and replacement of the sheet would have disturbed the protective microclimate and altered the curing conditions. These slabs were therefore inspected after hardening. A similar methodological limitation applied to part of the W6 slabs protected with the membrane-forming compound, for which only final crack inventory was obtained. Accordingly, final crack count and crack-development kinetics are distinguished throughout the analysis. Future full-scale studies should supplement visual crack counts with optical microscopy or digital image processing to quantify crack width, total length, cracked area, and spatial distribution, consistent with the more comprehensive crack-characterization philosophy used in standardized plastic-shrinkage testing such as ASTM C1579 [34].

### 2.11. Assessment of Evaporation Rate

The potential rate of water loss from the exposed surface of fresh concrete was assessed using the recorded environmental parameters, including air temperature, concrete temperature, relative humidity, and wind velocity. The evaporation rate E was estimated using the relationship proposed by Uno [1]:

E = 5([Tc + 18]2.5 − r[Ta + 18]2.5) (V + 4) × 10^−6^ (1)

where

E—evaporation rate [kg/m^2^/h];

Tc—concrete temperature [°C];

Ta—air temperature [°C];

r—relative humidity expressed as a decimal fraction;

V—wind velocity [km/h].

The evaporation-rate assessment was used to support interpretation of differences in cracking behavior between environmental exposure scenarios.

### 2.12. Statistical Analysis

The experimental data were analyzed using descriptive statistics and exploratory linear regression, with the statistical approach adapted to the structure and number of available observations. For compressive strength, results from three 150 mm × 150 mm × 150 mm cube specimens per mixture were expressed as mean ± SD (*n* = 3). Given this small replicate number, small between-mixture differences are interpreted cautiously and the strength results are not treated as a formal conformity assessment. For fresh concrete properties, relative slump loss after 60 min was calculated. Exploratory linear regressions were used only to describe associations between actual w/c ratio and (i) relative slump loss and (ii) 28-day cube compressive strength. For slump loss, W3 was excluded because of unintended water ingress, giving *n* = 6; the fitted relationship was y = 70.41(w/c) − 25.06, R^2^ = 0.817, *p* = 0.013, standard error of estimate = 3.76 percentage points, with a 95% confidence interval for the slope of 24.15 to 116.68. For compressive strength, all seven field mixtures were retained descriptively (*n* = 7); the fitted relationship was y = 78.39 − 64.39(w/c), R^2^ = 0.853, *p* = 0.003, standard error of estimate = 2.75 MPa, with a 95% confidence interval for the slope of −95.15 to −33.63 MPa per unit w/c. These regressions do not estimate an independent causal effect of w/c because admixture system, mixture composition, and production conditions were not held constant. Plastic shrinkage was analyzed using visible crack count. No inferential statistical comparisons were performed for surface-finishing technique, reinforcement configuration, or water-curing initiation time because only one full-scale slab represented each respective condition. No formal statistical ranking of curing methods was performed because group sizes and crack-monitoring protocols differed. Regression results and all case comparisons are therefore interpreted as descriptive associations rather than causal effects.

## 3. Results

### 3.1. Fresh Concrete Workability and Slump Retention

The initial slump values of the investigated concrete mixtures ranged from 140 to 160 mm, indicating that all mixtures exhibited a broadly comparable initial workability. However, considerable differences were observed in their ability to retain consistency over the subsequent 60 min. The measured slump values and the corresponding slump losses are summarized in Table 4.

The reference mixture W1 exhibited an initial slump of 155 mm, which decreased to 125 mm after 60 min, corresponding to a relative loss of 19.3%. Elimination of the superplasticizer in W2 slightly increased the slump loss to 22.6%. A substantially greater reduction in workability was observed for W4, in which the slump decreased from 155 to 110 mm, corresponding to a loss of 29.0%. The most pronounced loss of workability occurred for W5, produced without any water-reducing admixture. Despite having the highest actual w/c ratio (0.85), its slump decreased from 160 mm immediately after production to 100 mm after 60 min, representing a 37.5% reduction. This result indicates that increasing the mixing-water content was insufficient to provide effective long-term workability retention in the absence of a chemical admixture. In contrast, W7, containing the PCE-based MC-TechniFlow 25 superplasticizer, showed the highest workability retention among the correctly produced mixtures. Its slump decreased by only 25 mm, from 160 to 135 mm, corresponding to a relative reduction of 15.6%. W3 exhibited no measurable slump loss during the 60-min period. However, this result should not be interpreted as superior performance of the reduced-admixture formulation. As described in Section 2.2, unintended water ingress occurred during production of this mixture. Consequently, the W3 result represents an abnormal field-production condition rather than the intrinsic workability-retention behavior of the nominal formulation.

**Figure 4:**
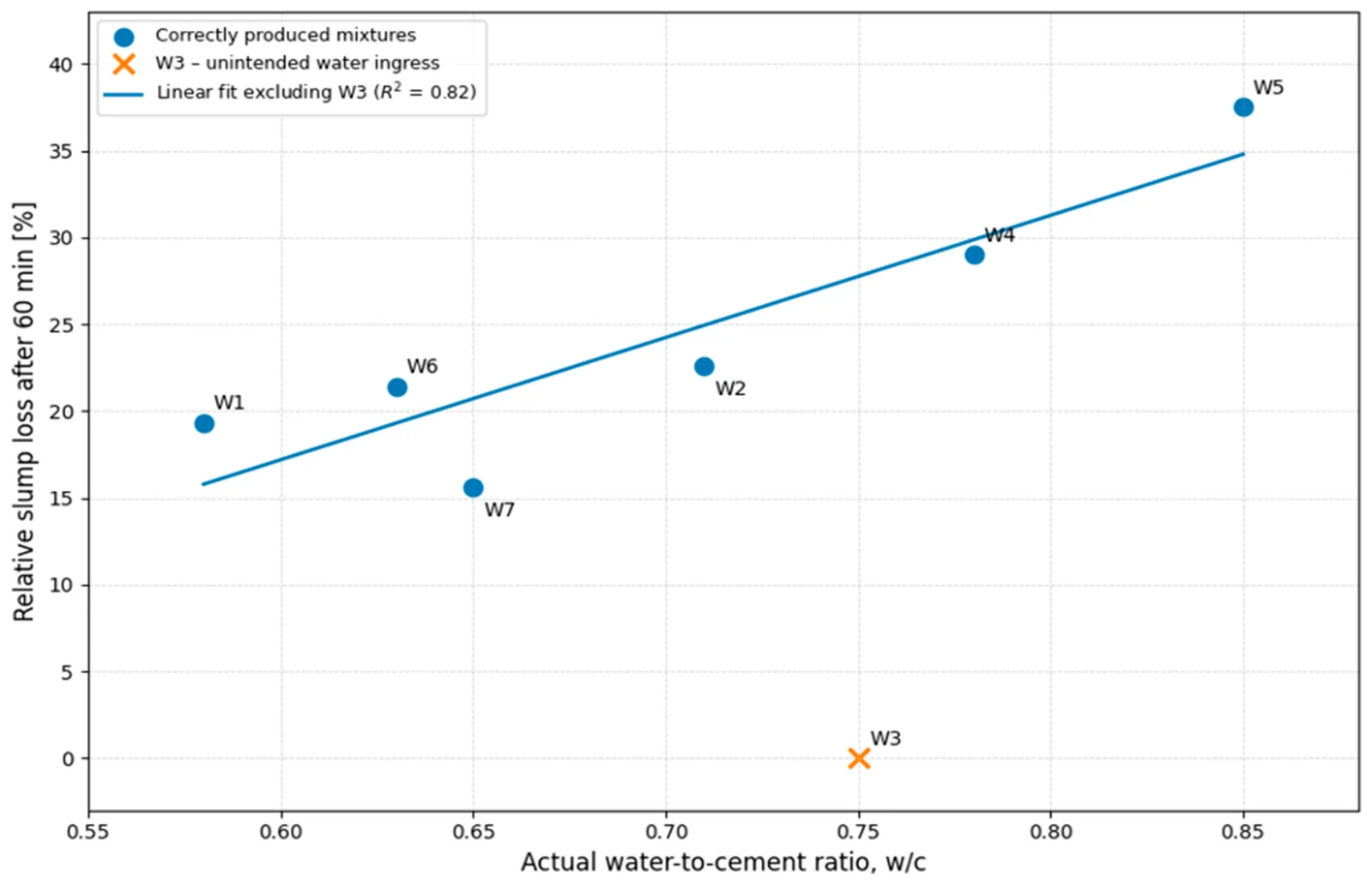
Relationship between the actual water-to-cement ratio and relative slump loss after 60 min. W3, affected by unintended water ingress during production, was treated as a technological anomaly and excluded from the regression analysis.

Figure 4 illustrates the relationship between the actual water-to-cement ratio and the relative slump loss after 60 min. For the correctly produced mixtures, an overall increase in slump loss was observed with increasing w/c ratio. The reference mixture W1, with the lowest w/c ratio of 0.58, exhibited a relative slump loss of 19.3%, whereas W5, produced without water-reducing admixtures and characterized by the highest w/c ratio of 0.85, showed the greatest loss of workability, reaching 37.5%. In contrast, W7, containing the PCE-based MC-TechniFlow 25 superplasticizer, exhibited a relatively low slump loss of 15.6% at a w/c ratio of 0.65, indicating improved workability retention compared with the other mixtures.

When W3 was excluded because of the documented unintended water ingress, an exploratory positive association was observed between actual w/c ratio and relative slump loss for the six correctly produced mixtures: relative slump loss = 70.41(w/c) − 25.06 (*n* = 6, R^2^ = 0.817, *p* = 0.013; 95% CI for slope: 24.15–116.68). The standard error of estimate was 3.76 percentage points. This relationship is descriptive only, because each mixture also represented a different admixture type and/or dosage; consequently, the apparent association cannot be interpreted as evidence that w/c independently controls slump retention. W3 showed no measurable slump loss despite w/c = 0.75, but this behavior is not evidence of superior rheological stability because the mixture was affected by an unplanned field-production event involving unintended water ingress. W3 is therefore retained only as a practically relevant anomaly and not as evidence of a systematic effect of reduced admixture dosage or finishing procedure.

Overall, the results demonstrate that initial consistency alone was not sufficient to characterize the workability performance of the investigated concretes. Mixtures with similar initial slump values showed substantially different slump retention after 60 min, highlighting the important role of the admixture system in maintaining workability under conditions representative of prolonged ready-mixed concrete transport.

### 3.2. Compressive Strength

The compressive strength results obtained after 28 days are presented in Table 5. For each concrete mixture, three 150 mm × 150 mm × 150 mm cube specimens were tested, and the results are expressed as mean ± standard deviation (*n* = 3). All mixtures had been designed for strength class C30/37; however, the present results do not constitute a formal conformity assessment according to EN 206+A2:2021-08 [35]. In view of the small replicate number, differences between close mean values should be interpreted cautiously.

The reference concrete W1, characterized by the lowest actual w/c ratio of 0.58 and containing the complete admixture system, achieved the highest mean compressive strength of 41.4 MPa. Reducing the effectiveness or amount of the water-reducing admixtures generally increased the amount of water required to maintain the target consistency and was accompanied by a marked decrease in 28-day compressive strength. For W2, an increase in the actual w/c ratio from 0.58 to 0.71 resulted in a reduction in mean compressive strength from 41.4 to 33.4 MPa, corresponding to a decrease of approximately 19.3% relative to W1. A further deterioration was observed for mixtures characterized by higher water contents. W4, with an actual w/c ratio of 0.78, achieved a mean compressive strength of 30.8 MPa, approximately 25.6% lower than the reference mixture. The most pronounced reduction was recorded for W5, in which water-reducing admixtures were completely eliminated. Maintaining the required initial workability required an increase in the actual w/c ratio to 0.85, and the resulting mean compressive strength decreased to 24.6 MPa, representing a reduction of approximately 40.6% compared with W1. Thus, the additional mixing water used to compensate for the absence of chemical admixtures enabled the target fresh-concrete consistency to be achieved but substantially impaired the mechanical performance of the hardened concrete.

The W3 mixture also exhibited a very low mean compressive strength of 24.7 MPa. However, this result requires separate interpretation because unintended water ingress occurred during production. Consequently, W3 should be regarded as a technological anomaly rather than as a mixture representing only the nominal reduction in admixture dosage. The very low strength obtained for W3 nevertheless illustrates the potentially severe mechanical consequences of uncontrolled additional water introduced into fresh concrete. A different response was observed for W6 and W7. Increasing the cement content in W6 enabled a mean compressive strength of 38.3 MPa to be obtained despite an actual w/c ratio of 0.63. W7, incorporating the PCE-based MC-TechniFlow 25 superplasticizer, reached 36.8 MPa at a w/c ratio of 0.65. These results were considerably higher than those obtained for W2–W5, demonstrating that maintaining workability through an appropriate admixture system was substantially more effective than compensating for reduced admixture efficiency by increasing the mixing-water content.

**Figure 5:**
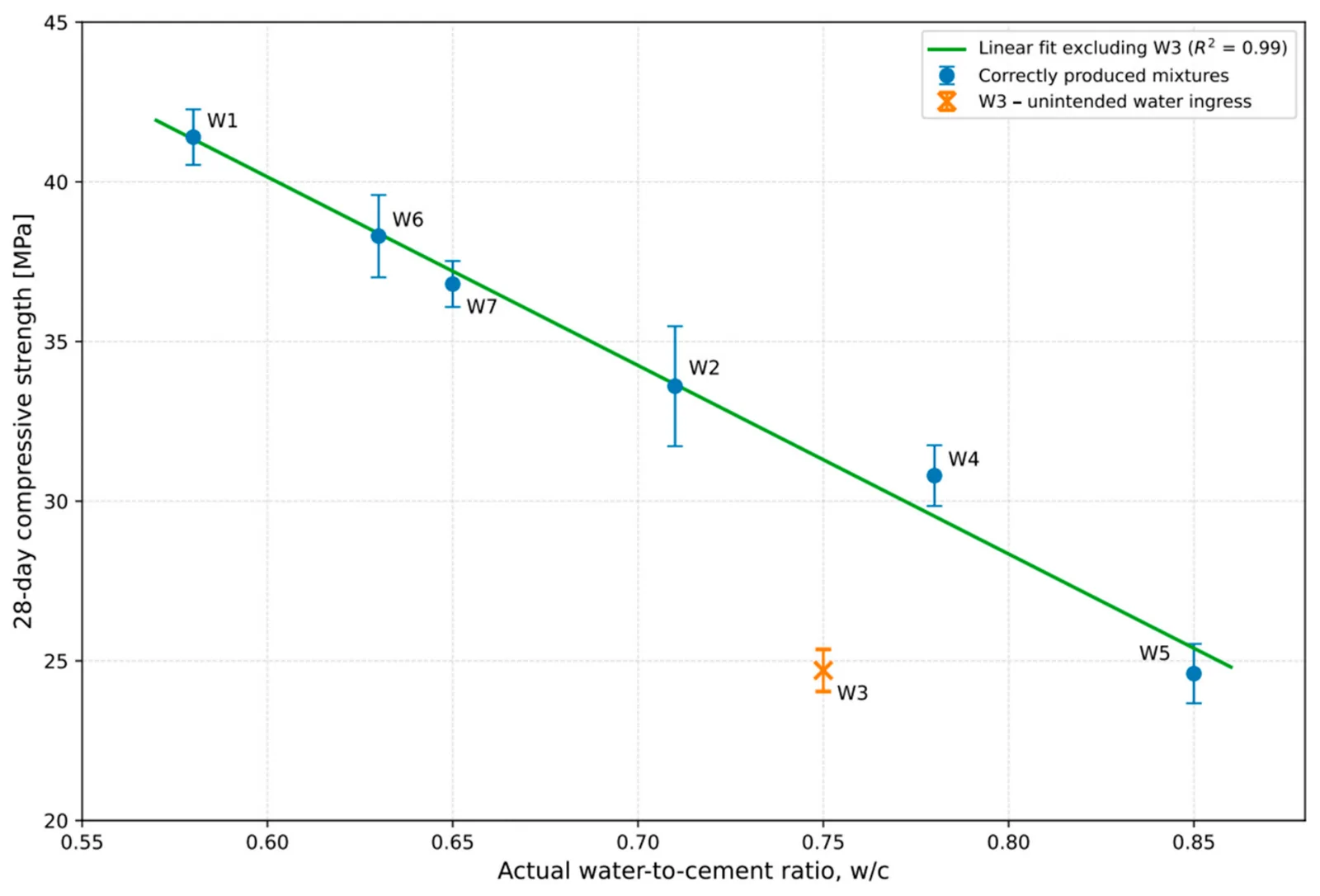
Relationship between the actual water-to-cement ratio and 28-day compressive strength of the investigated concrete mixtures. Error bars represent standard deviations (*n* = 3). W3 was affected by unintended water ingress during production.

Figure 5 illustrates the relationship between the actual w/c ratio and 28-day compressive strength. The overall results indicate a pronounced reduction in compressive strength with increasing w/c ratio. In particular, the transition from W1 (w/c = 0.58) to W5 (w/c = 0.85) was associated with a reduction in mean compressive strength from 41.4 to 24.6 MPa. When the obtained 28-day cube strengths were compared with the target strength level corresponding to C30/37 concrete, W1 showed the most favorable performance, whereas the remaining mixtures exhibited lower mean strength values. These results should not be interpreted as a formal conformity assessment according to EN 206+A2:2021-08 [35], because the experimental sampling programme was not designed for production conformity evaluation.

Overall, the results demonstrate that reducing or eliminating water-reducing admixtures while maintaining a similar initial consistency by increasing the mixing-water content resulted in a substantial loss of compressive strength. The findings therefore emphasize that equivalent initial workability does not imply equivalent hardened-concrete performance, particularly when the target consistency is obtained through an increase in the water-to-cement ratio rather than through effective particle dispersion by chemical admixtures.

### 3.3. Plastic Shrinkage Cracking of Full-Scale Slabs

Plastic shrinkage cracking showed substantial variation among the investigated full-scale slabs, demonstrating that early-age cracking was strongly dependent on the combined technological and environmental conditions prevailing during concrete placement and the first hours after casting. The observed response ranged from complete absence of visible cracking in effectively protected slabs to extensive crack development in unprotected elements exposed to unfavorable conditions. A clear distinction was observed between slabs without surface protection and those subjected to an effective curing procedure. Unprotected slabs, including those produced using mixtures W2–W5 and the unprotected reference slab W1-P2, developed visible surface cracking. Depending on the mixture, environmental exposure, surface-finishing procedure, and reinforcement configuration, the final number of recorded cracks ranged from 3 to 19 per slab. The most severe cracking was observed for the W4 series exposed to the highest air temperatures and lowest relative humidity recorded during the experimental program.

For W4-P1, the first visible cracks appeared after approximately 90 min, when five cracks were recorded. Their number subsequently increased to 8 after 120 min, 11 after 150 min, 14 after 180 min, 16 after 210 min, and finally 19 cracks after 240 min. The corresponding W4-P2 slab exhibited a similar onset of cracking but a lower subsequent rate of crack development. Four cracks were recorded after 90 min, increasing to 7 after 120 min, 10 after 150 min, 12 after 180 min, and 13 after 240 min. These results indicate that, once cracking was initiated, the number of visible cracks could increase rapidly during the subsequent 2–3 h when the concrete surface remained exposed to drying. The first 4 h after casting therefore represented a critical period for the development of plastic shrinkage cracking in the investigated full-scale elements. In contrast, substantially lower crack counts were observed under moderate environmental conditions. For example, W5-P1, cast at an air temperature of approximately 22.1 °C under cloudy conditions, developed only two visible cracks after 90 min and four cracks after 150 min. No further increase was observed up to 240 min. In comparison, W4-P1, exposed to temperatures reaching 35.2 °C, developed 19 cracks over the same observation period. The results also showed that effective surface protection could markedly limit or completely prevent visible plastic shrinkage cracking. Water-cured W6 slabs exhibited very limited cracking, with a maximum of only one recorded crack, while several slabs protected using barrier-type curing procedures remained free of visible cracks.

The overall observations therefore demonstrate that the cracking response of the full-scale slabs cannot be attributed solely to concrete mixture composition. Although mixture design and water content affected fresh and hardened concrete properties, the development of plastic shrinkage cracking was strongly modified by environmental exposure, reinforcement configuration, surface finishing, and—most importantly—the effectiveness and timing of curing.

**Figure 6:**
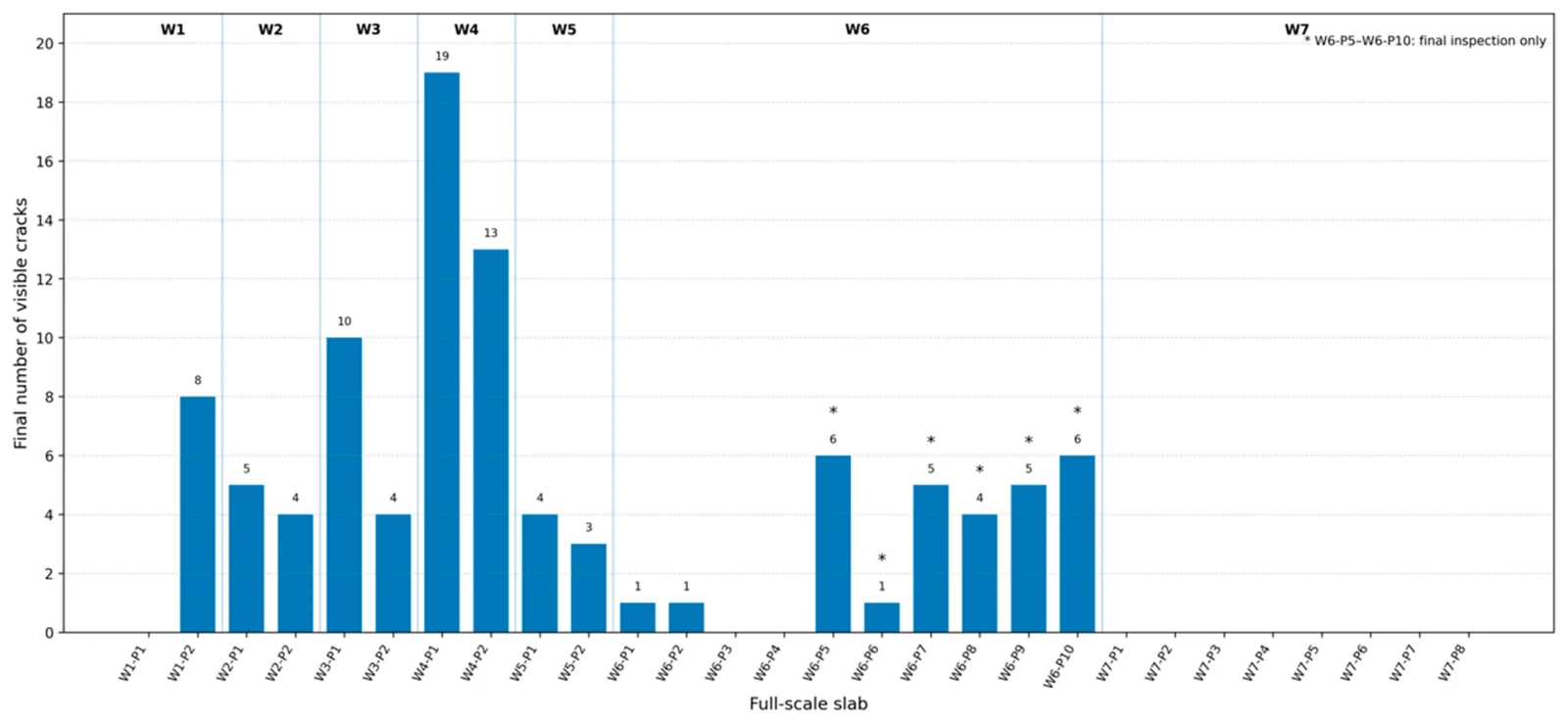
Final number of visible plastic shrinkage cracks recorded for the 28 full-scale concrete slabs. For * W6-P5–W6-P10, only the final crack count was recorded after setting.

Figure 6 provides an overall comparison of the final number of visible plastic shrinkage cracks recorded for all 28 full-scale concrete slabs.

A pronounced differentiation in cracking behavior was observed depending on the surface-protection strategy and the technological and environmental conditions prevailing during casting and early hardening. The unprotected slabs exhibited the highest susceptibility to plastic shrinkage cracking. In this group, the final crack count ranged from 3 to 19 cracks per slab. Particularly severe cracking occurred in the W4 series. W4-P1, reinforced with Ø12 mm bars, developed 19 cracks, while W4-P2, containing the standard Ø6 mm reinforcement, developed 13 cracks. These were the highest values recorded in the entire experimental program. Substantial cracking was also observed for W3-P1, finished using a vibrating screed, for which 10 cracks were recorded, compared with only 4 cracks for W3-P2, finished manually. The unprotected reference slab W1-P2 developed 8 cracks, whereas W1-P1, protected immediately with the membrane-forming curing compound, remained completely free of visible cracking. A particularly clear effect of surface protection was observed for the W7 series. All eight W7 slabs were protected using PE sheeting, and no visible plastic shrinkage cracks were recorded on any of these elements. This result demonstrates the high effectiveness of an impermeable physical barrier in limiting moisture loss from the fresh concrete surface during the critical early-age period. The results obtained for W6 provide additional information on the practical effectiveness of different curing procedures.

The slabs subjected to water curing or effectively protected with PE sheeting exhibited zero or only one visible crack. In contrast, some slabs treated with the membrane-forming curing compound developed between 1 and 6 cracks. For W6-P5–W6-P10, only the final crack count was recorded after setting; therefore, these results describe the final cracking state rather than the kinetics of crack development.

Overall, Figure 6 demonstrates that surface protection was one of the dominant factors controlling plastic shrinkage cracking in the full-scale slabs. Nevertheless, the variation observed among the unprotected elements indicates that curing was not the only governing parameter. Environmental exposure, surface-finishing technique, reinforcement configuration, and mixture-related factors also contributed to the observed cracking response. These effects are analyzed separately in the following sections.

### 3.4. Effect of Surface Finishing on Plastic Shrinkage Cracking

**Figure 7:**
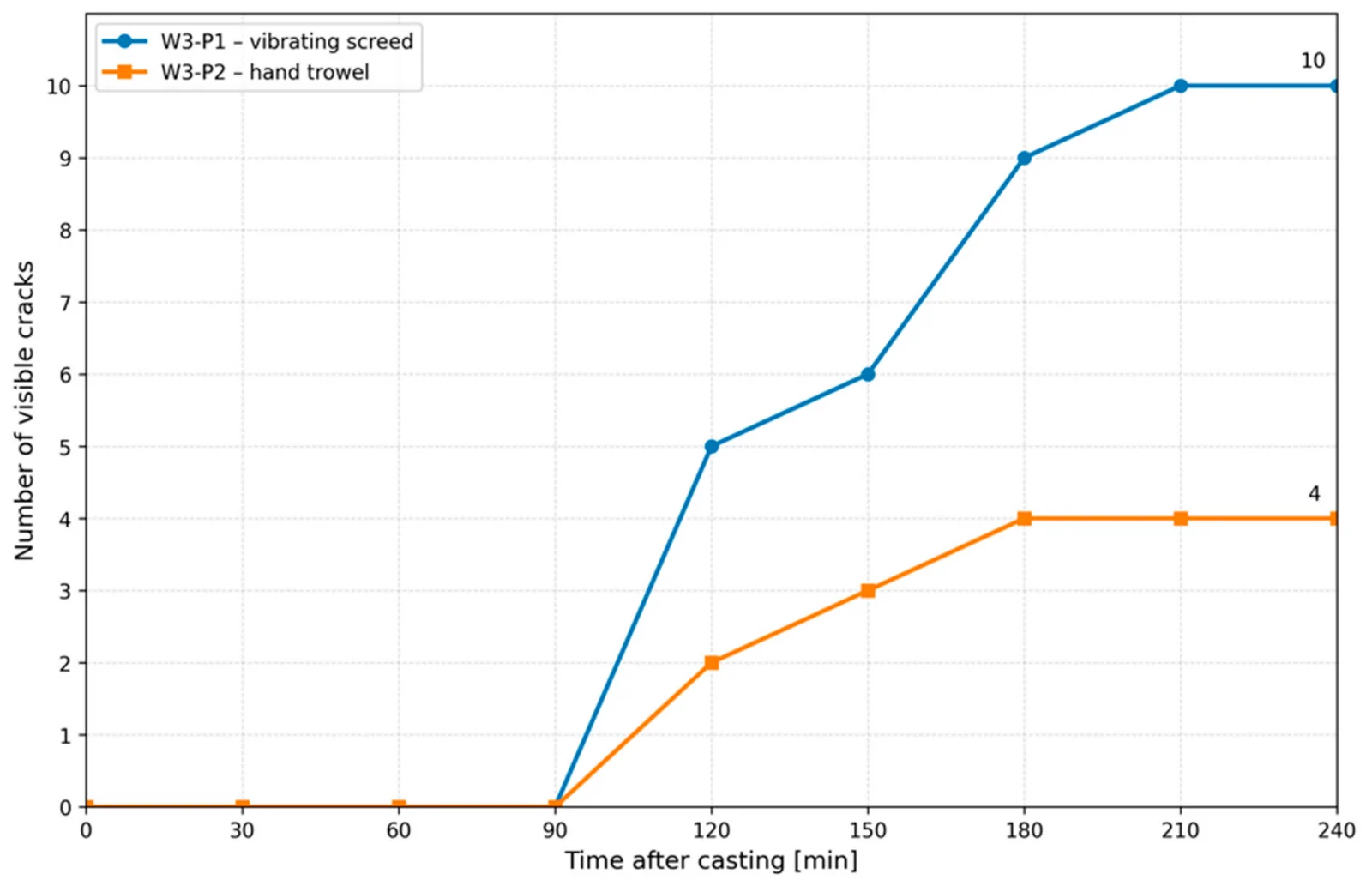
Development of the number of visible plastic shrinkage cracks over time for W3 slabs finished using a vibrating screed (W3-P1) and a hand trowel (W3-P2).

Two full-scale slabs produced from the same W3 concrete batch were finished differently: W3-P1 using a vibrating screed and W3-P2 using a steel hand trowel. However, W3 had been affected by unintended water ingress during production and therefore cannot be regarded as a controlled mixture variant. In addition, only one slab represented each finishing condition. The comparison is consequently presented only as a case-specific field observation and is not used to infer a general or statistically supported effect of finishing technique. The observed crack counts are summarized in Table 6 and Figure 7.

The evolution of the number of visible cracks during the first 240 min after casting is presented in Table 6.

No visible cracks were observed on either slab during the first 90 min after casting. The first cracks were recorded after 120 min, when W3-P1 exhibited five cracks compared with two cracks on W3-P2. Thereafter, the difference between the slabs increased progressively. After 180 min, nine cracks were observed on the surface finished with the vibrating screed, compared with four cracks on the manually finished slab. At the end of the 240-min observation period, W3-P1 exhibited 10 visible cracks, whereas only 4 cracks were recorded on W3-P2. Thus, under the conditions of this experiment, the final crack count for the vibrating-screed-finished slab was 2.5 times higher than that observed for the manually finished slab.

The difference between the two slabs became apparent immediately after crack initiation and persisted throughout the remaining observation period. The crack count on W3-P1 continued to increase until 210 min, whereas no further increase was recorded for W3-P2 after 180 min. Consequently, the manually finished surface exhibited not only a lower final crack count but also an earlier stabilization of crack development. The W3 mixture was affected by unintended water ingress during production, as described in Section 2.2. This factor may have increased the overall susceptibility of the concrete to plastic shrinkage cracking. However, because both W3-P1 and W3-P2 were produced from the same mixture, the comparison between these two slabs remains useful for evaluating the relative effect of the surface-finishing procedure under the specific conditions of the experiment. The results therefore indicate that the surface-finishing method can significantly modify the development of early-age cracking even when concrete composition and environmental exposure are nominally the same. However, because the comparison was based on one full-scale slab per finishing method, the observed difference should be interpreted as a case-specific experimental result rather than as a statistically generalizable effect.

### 3.5. Effect of Reinforcement Configuration on Plastic Shrinkage Cracking

The influence of reinforcement configuration on plastic shrinkage cracking was evaluated using two full-scale slabs produced from the W4 concrete mixture. Both slabs were cast under the same environmental conditions and subjected to the same surface-finishing and exposure procedures, while differing in reinforcement configuration. W4-P1 contained a single reinforcement mesh made of Ø12 mm bars, whereas W4-P2 contained the standard double mesh made of Ø6 mm bars. The W4 series was cast under the most severe environmental conditions encountered during the experimental programme, with air temperatures ranging from 33.4 to 35.2 °C and relative humidity of only 28–30%. These conditions resulted in rapid surface drying and provided a particularly demanding scenario for evaluating the effect of reinforcement configuration on early-age cracking. The development of visible cracks during the first 240 min after casting is presented in Table 7.

Visible cracking developed earlier in the W4 slabs than in the W3 series discussed in Section 3.4. The first cracks were recorded after 90 min, with five cracks observed on W4-P1 and four on W4-P2. Crack development continued throughout the entire observation period for both slabs. The difference between the two reinforcement configurations became progressively more pronounced with time. After 150 min, 11 cracks were recorded on W4-P1 compared with 10 on W4-P2. After 180 min, the corresponding values increased to 14 and 12 cracks, respectively. At the end of the 240-min observation period, W4-P1 exhibited 19 visible cracks, whereas W4-P2 exhibited 13 cracks. Thus, the final crack count for the slab reinforced with Ø12 mm bars was approximately 46% higher than that recorded for the slab containing the standard Ø6 mm reinforcement configuration.

**Figure 8:**
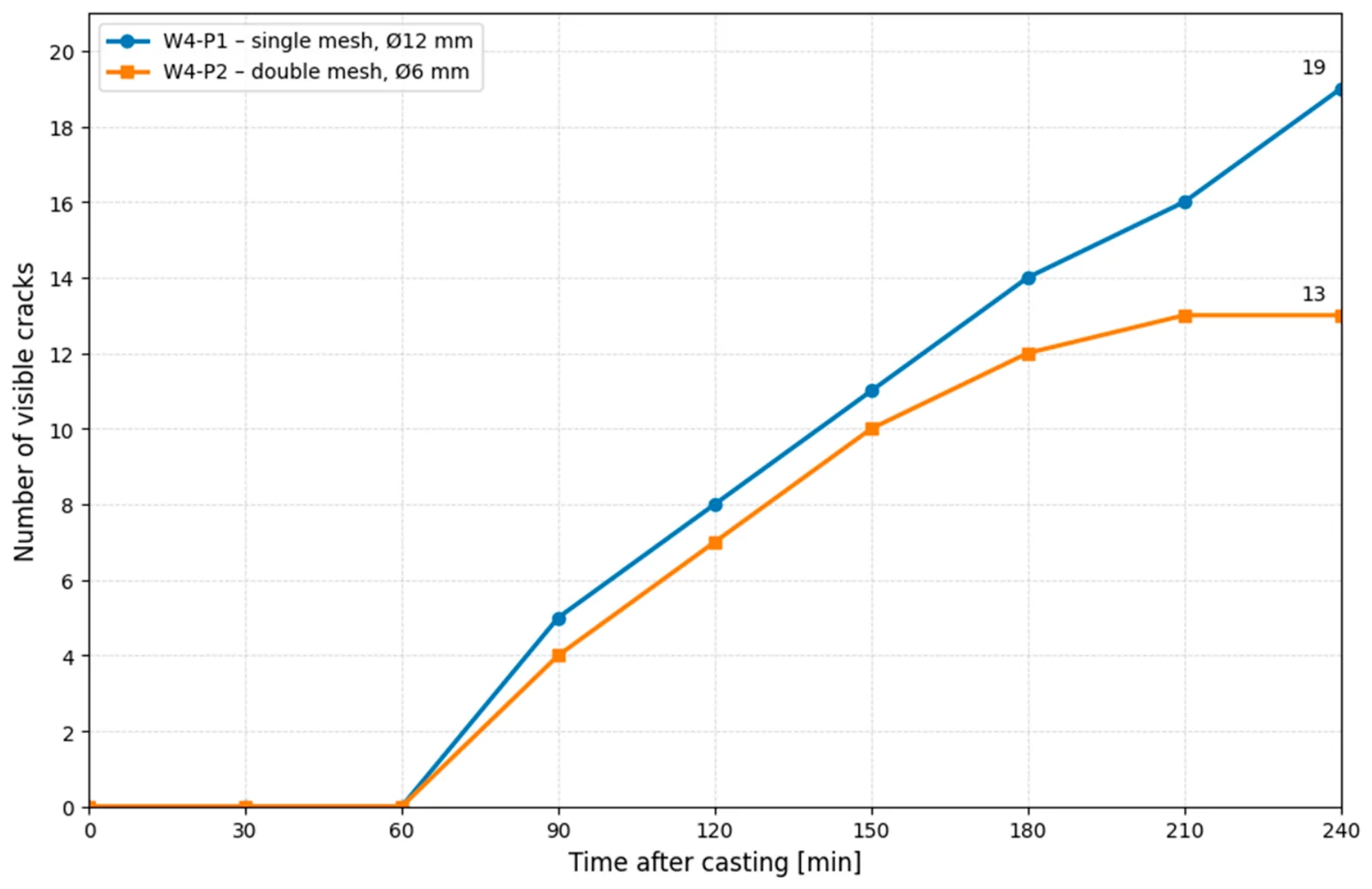
Development of visible plastic shrinkage cracks over time for W4 slabs with different reinforcement configurations: W4-P1 (Ø12 mm) and W4-P2 (Ø6 mm).

The temporal evolution of cracking indicates that the reinforcement configuration did not substantially change the time of crack initiation, since visible cracking was first recorded after 90 min for both slabs—Figure 8. Instead, the principal difference concerned the subsequent development of cracking. The number of cracks continued to increase more rapidly in W4-P1, particularly during the later part of the observation period. The observed behavior suggests that the reinforcement configuration influenced the restraint conditions within the near-surface concrete and consequently affected the development of plastic shrinkage cracking. The larger-diameter reinforcement used in W4-P1 was associated with a higher final crack count than the standard Ø6 mm reinforcement arrangement under otherwise comparable conditions. However, as in the comparison of surface-finishing methods, the results should be interpreted with caution. Only one full-scale slab was investigated for each reinforcement configuration, and the W4 series was exposed to particularly severe drying conditions. Therefore, the results demonstrate a clear difference between the two investigated slabs but should not be interpreted as establishing a general quantitative relationship between bar diameter and plastic shrinkage cracking.

### 3.6. Effect of Environmental Exposure on Plastic Shrinkage Cracking

The influence of environmental exposure on plastic shrinkage cracking was assessed by comparing the cracking behaviour observed under markedly different weather conditions. Particular attention was given to the W4 and W5 series, which represented severe hot-weather exposure and substantially more moderate conditions, respectively. During casting of W4, the air temperature ranged from 33.4 to 35.2 °C, while the relative humidity was only 28–30% and the wind velocity ranged from 1.6 to 2.2 m/s. In contrast, W5 was cast at an air temperature of approximately 21.9–22.1 °C, relative humidity of 50–53%, and wind velocity of 1.1–1.4 m/s. Thus, the W4 series was exposed to substantially more severe drying conditions. The development of visible cracks for representative unprotected slabs is summarized in Table 8.

Visible cracking was first detected after 90 min in both slabs, but its subsequent development differed markedly. At this time, W4-P1 already exhibited five cracks compared with two cracks in W5-P1. After 150 min, the crack count increased to 11 for W4-P1 and to only four for W5-P1. No additional cracks were observed in W5-P1 between 150 and 240 min, whereas cracking in W4-P1 continued throughout the entire observation period and reached 19 cracks after 240 min.

**Figure 9:**
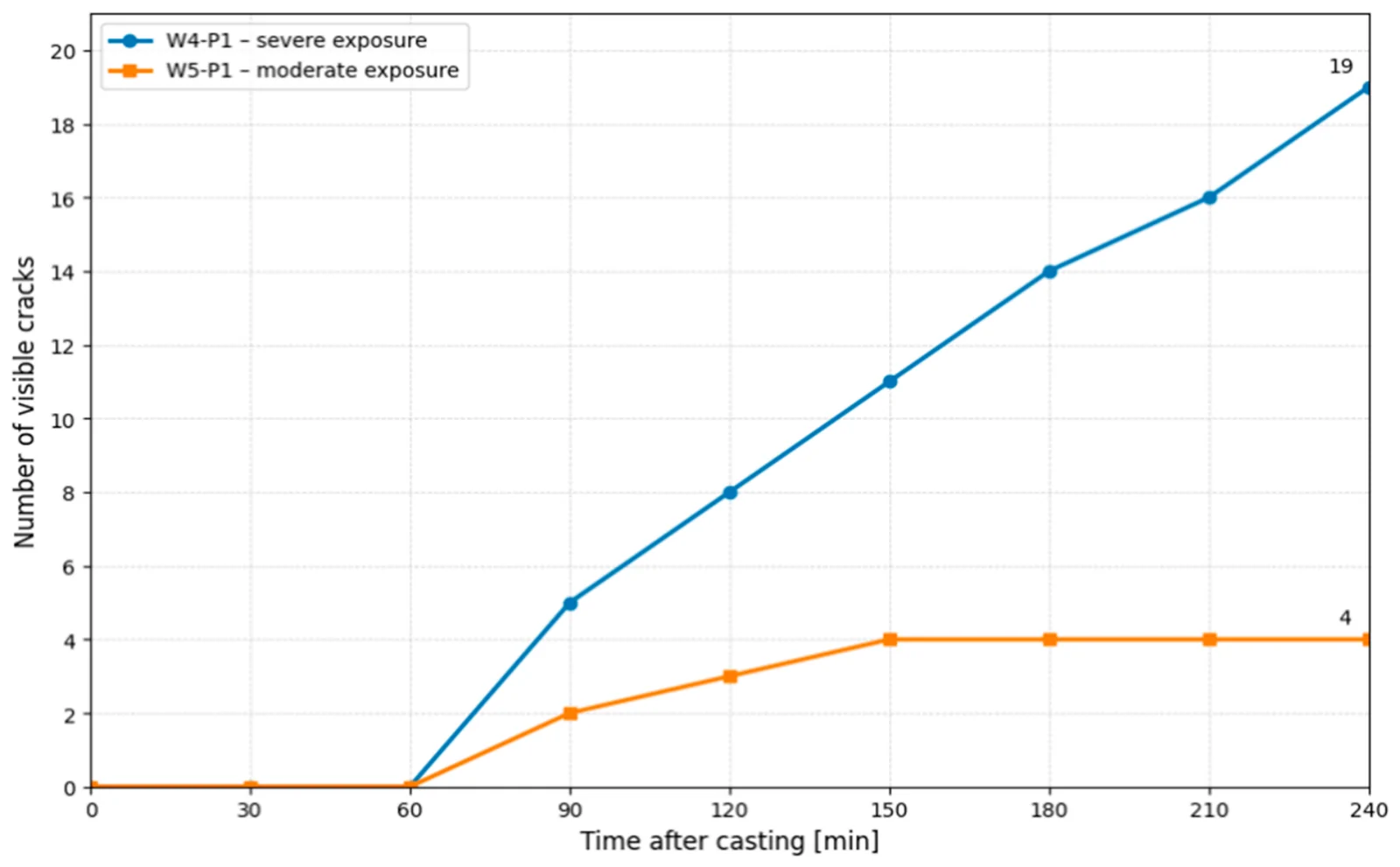
Development of visible plastic shrinkage cracks over time for representative unprotected slabs exposed to severe (W4-P1) and moderate (W5-P1) environmental conditions.

At the end of the observation period, W4-P1 had 19 visible cracks and W5-P1 had 4. The W4 series was associated with substantially hotter and drier exposure than W5, and crack development continued throughout the W4 observation period while the W5 count stabilized after approximately 150 min—Figure 9. However, this comparison is not a controlled single-variable experiment: W4 and W5 differed in mixture composition, actual w/c ratio, admixture system, and reinforcement configuration. Therefore, the 19-versus-4 difference cannot be attributed solely to environmental conditions. It is more appropriately interpreted as evidence that the severe W4 field-production scenario was associated with substantially greater visible cracking than the more moderate W5 scenario.

However, the W4–W5 comparison should not be interpreted as a controlled single-variable experiment. The two mixtures also differed in composition and actual w/c ratio, and W4-P1 contained the alternative reinforcement configuration. Therefore, the observed differences represent the combined response of full-scale concrete elements under different production and environmental scenarios. Nevertheless, the much greater and sustained crack development observed during the W4 experiment indicates that severe hot and dry exposure was an important factor contributing to the susceptibility of the concrete surface to plastic shrinkage cracking.

### 3.7. Effectiveness of Curing Methods in Limiting Plastic Shrinkage Cracking

**Figure 10:**
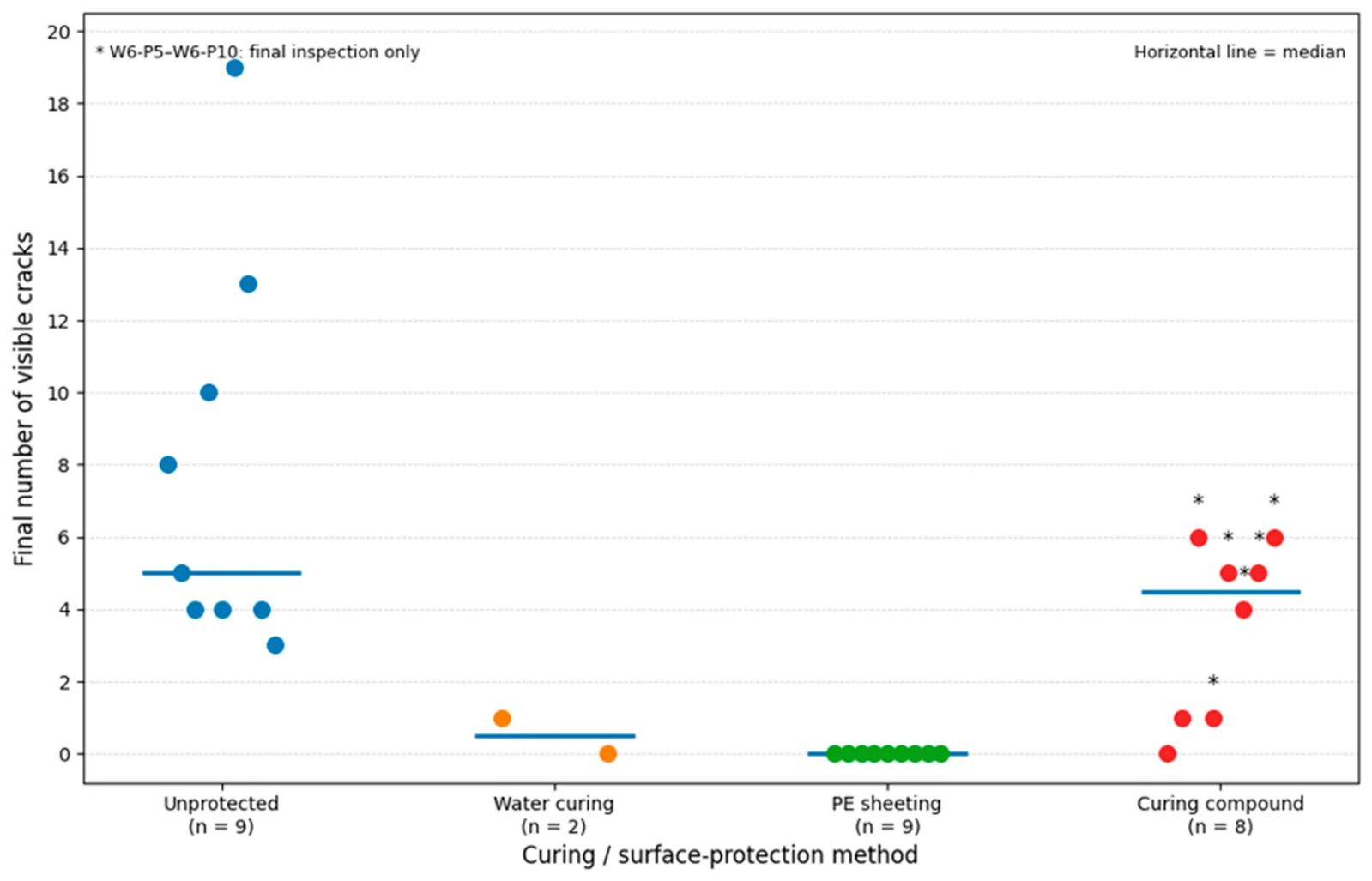
Final number of visible plastic shrinkage cracks in full-scale slabs subjected to different curing and surface-protection methods. Individual points represent individual slabs, while horizontal lines indicate median values. For * W6-P5–W6-P10, only the final crack count was recorded after setting.

The effectiveness of different curing and surface-protection methods was evaluated using the full-scale slabs, with particular emphasis on the extended W6 and W7 series. The investigated procedures included water curing, polyethylene (PE) sheeting, application of the CHRYSO Cure HPP membrane-forming curing compound, and, for comparison, exposure without surface protection. A clear reduction in plastic shrinkage cracking was observed when moisture loss from the fresh concrete surface was effectively limited. The most consistent performance was obtained with PE sheeting. W6-P3, protected with PE sheeting, remained completely free of visible cracks. Similarly, all eight W7 slabs protected with PE sheeting exhibited no visible cracking in the final inspection. For slabs protected with PE sheeting, continuous 30-min monitoring was intentionally not performed because repeated removal of the covering would have disturbed the protective microclimate. These elements were therefore inspected after hardening. Water curing also resulted in very limited cracking. W6-P1, for which water curing was initiated as soon as the surface condition allowed application, developed only one visible crack, whereas W6-P4, for which water curing was initiated after 90 min, remained free of visible cracks. Because only one slab represented each water-curing timing condition, these results do not provide sufficient evidence to establish a quantitative relationship between curing initiation time and crack development. They do, however, demonstrate that both water-curing procedures were associated with very low crack counts under the investigated conditions. The performance of the membrane-forming curing compound was more variable. W1-P1, protected with CHRYSO Cure HPP, remained free of visible cracking, while W6-P2 developed only one crack. In contrast, the final crack counts recorded for W6-P5–W6-P10 were 6, 1, 5, 4, 5, and 6, respectively. For W6-P5–W6-P10, only the final crack count was recorded after setting rather than through continuous 30-min monitoring. This methodological difference should therefore be considered when comparing these slabs with specimens subjected to full-time observation—Figure 10.

Overall, the results indicate that physical isolation of the concrete surface using PE sheeting provided the most consistent prevention of visible plastic shrinkage cracking within the investigated program. Water curing also resulted in very low crack counts. The membrane-forming compound showed greater variability, ranging from complete crack prevention to several visible cracks per slab. This variability should not be interpreted solely as a material-related effect, because the effectiveness of membrane-forming curing compounds may also depend on application uniformity, timing, continuity of the protective film, and local exposure conditions. The original observations indicated that non-uniform application of the curing compound could reduce the continuity of the protective barrier. The results therefore support the conclusion that effective restriction of early surface moisture loss was a key factor in reducing plastic shrinkage cracking, although the individual curing methods cannot be ranked statistically because the numbers of slabs assigned to each condition were unequal and not all specimens were monitored using the same observation protocol.

### 3.8. Evaporation Conditions and Plastic Shrinkage Cracking

The environmental conditions recorded during the full-scale experiments showed substantial differences in the calculated potential for moisture loss from the fresh concrete surface. The most pronounced contrast occurred between the W4 and W5 field-production scenarios. During W4, the maximum air temperature reached 35.2 °C, relative humidity decreased to approximately 28–30%, and wind velocity reached 2.2 m/s; the slabs were also exposed to direct solar radiation. W5 was cast under cloudy conditions at approximately 22.1 °C with a wind velocity of approximately 1.1 m/s. The evaporation rate was estimated using the Uno relationship described in Section 2.11; it was not measured directly as mass loss from the concrete surface. The calculated values are therefore referred to consistently as estimated or calculated potential evaporation rates. The W4 scenario was associated with more sustained visible crack development than W5, but because mixture and reinforcement variables also differed, the comparison does not isolate an independent environmental effect.

Therefore, individual environmental parameters should not be considered independently when assessing the risk of early-age cracking. The observations are particularly relevant for the investigated concretes because the binder system contained CEM II/B-M (S-V) cement and additional siliceous fly ash. In the source study, attention was drawn to the fact that conventional evaporation thresholds should be interpreted cautiously for concretes containing supplementary cementitious materials because their bleeding behavior may differ from that of conventional Portland-cement concrete. The experimental results further demonstrate that the consequences of severe drying conditions depended strongly on whether the concrete surface was protected. Although environmental exposure created the potential for rapid moisture loss, effective curing—particularly the use of PE sheeting and appropriately initiated water curing—substantially limited visible cracking, as demonstrated in Section 3.7. Thus, the environmental drying potential and the effectiveness of surface protection should be considered jointly when evaluating the risk of plastic shrinkage cracking.

Overall, the results indicate that the first hours after placement represented the critical period for moisture-loss-induced cracking. Under severe exposure, crack initiation occurred within approximately 90 min and continued throughout the 240-min observation period. This finding emphasizes the importance of implementing surface-protection measures before significant evaporation from the fresh concrete can occur.

**Figure 11:**
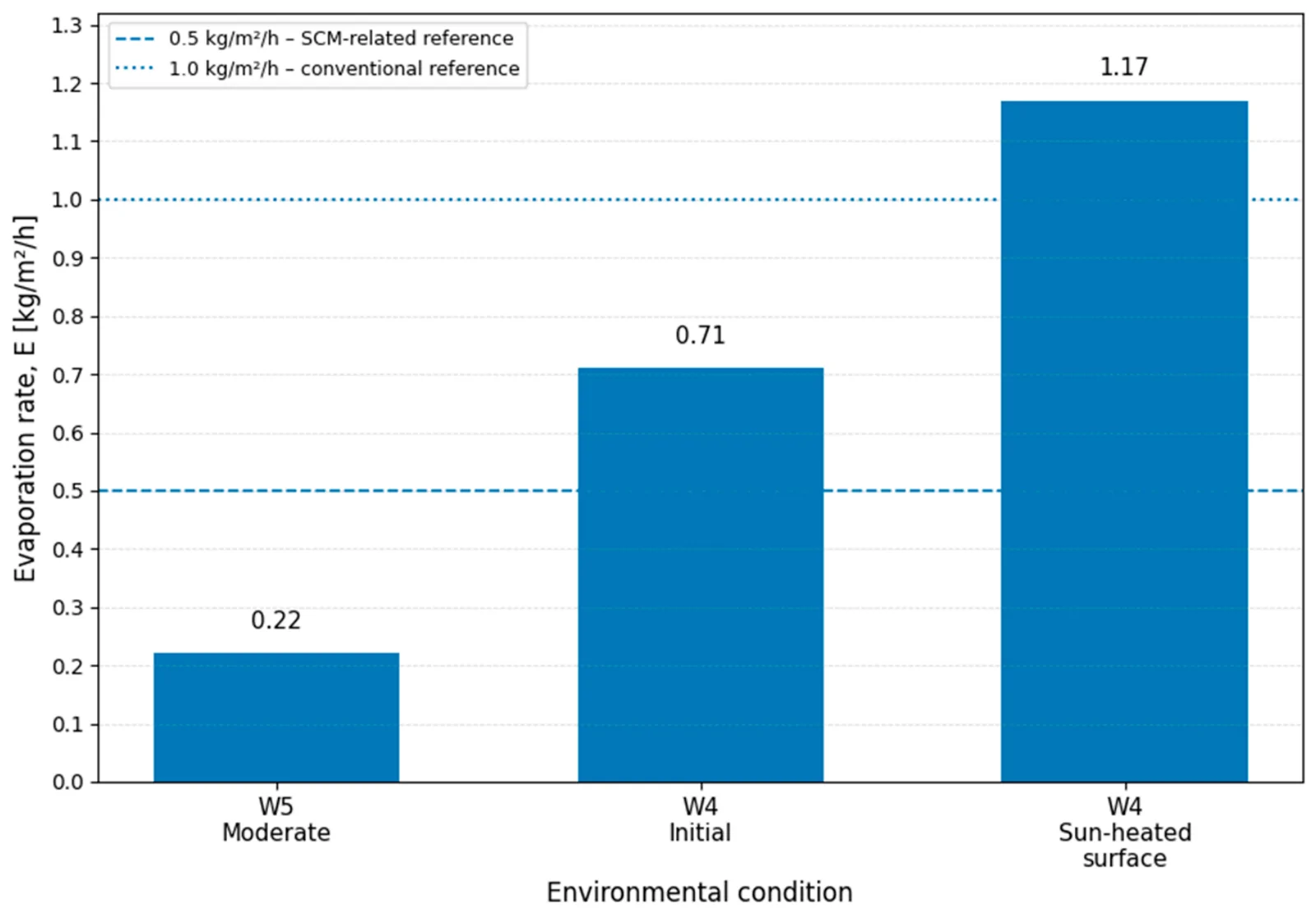
Evaporation rates calculated using the Uno model for moderate W5 conditions and severe W4 exposure, including the effect of solar heating of the concrete surface. Dashed reference lines indicate evaporation rates of 0.5 and 1.0 kg/m^2^/h.

The calculated potential evaporation rate was 0.22 kg/m^2^/h for the moderate W5 scenario and 0.71 kg/m^2^/h for the initial W4 conditions—Figure 11. An additional field estimate considered a concrete surface temperature of approximately 40 °C during direct solar heating, which yielded 1.17 kg/m^2^/h. Because the instrument metadata, exact measurement position/interval, and solar irradiance were not documented with sufficient detail for reproducible quantitative analysis, the 40 °C value and the corresponding 1.17 kg/m^2^/h result are treated only as an illustrative sensitivity scenario and not as a primary measured environmental variable. The principal interpretation is therefore based on the documented air temperature, relative humidity, wind velocity, and concrete-temperature ranges. The greater crack count observed for W4-P1 relative to W5-P1 is reported as an association between two different field-production scenarios, not as proof that environmental exposure independently caused the difference.

However, the relationship should not be interpreted as a direct quantitative correlation between evaporation rate and crack number, because W4 and W5 differed not only in environmental exposure but also in mixture composition and, for the selected slabs, reinforcement configuration. Nevertheless, the combined results provide clear experimental evidence that rapid moisture loss during the first hours after casting substantially increased the susceptibility of the exposed concrete surface to plastic shrinkage cracking.

## 4. Discussion

The results of this study demonstrate that plastic shrinkage cracking in full-scale concrete slabs is controlled by the interaction of mixture composition, fresh-concrete rheology, environmental drying potential, finishing procedures, reinforcement configuration, and early-age curing [1,3,8,11,19,21]. The full-scale observations are particularly important because most laboratory studies isolate only one or two of these variables, whereas actual ready-mixed concrete construction involves their simultaneous action. In the present program, 28 full-scale slabs were exposed to realistic production and environmental conditions. A particularly relevant outcome is the clear distinction between parameters governing hardened mechanical performance and those controlling plastic-stage cracking. Increasing the actual w/c ratio resulted in a pronounced decrease in compressive strength, whereas plastic shrinkage cracking was strongly modified by evaporation conditions and surface protection [1,8,10,21]. Consequently, neither w/c ratio nor compressive strength alone can be used as a reliable indicator of early-age cracking risk.

### 4.1. Influence of Mixture Design and Water-to-Cement Ratio

Progressive reduction or elimination of water-reducing admixtures required increasing amounts of mixing water to maintain the target initial consistency. The reference W1 mixture achieved an initial slump of 155 mm at an actual w/c ratio of 0.58, whereas the admixture-free W5 mixture required a w/c ratio of 0.85 to achieve a comparable initial consistency. This technological adjustment had substantial mechanical consequences. The 28-day mean compressive strength decreased from 41.4 MPa for W1 to 24.6 MPa for W5, corresponding to a reduction of approximately 40.6%. The results therefore confirm that achieving comparable initial workability through additional water is not equivalent to achieving it through effective dispersion of cement particles. This interpretation is consistent with the established mechanisms of water-reducing admixtures and modern polycarboxylate ether superplasticizers. PCEs improve cement-particle dispersion predominantly through steric effects, reducing the amount of water required to obtain a specified workability and contributing to improved workability retention [12,15,36,37,38]. Recent studies have also demonstrated that the molecular architecture of PCEs, including side-chain structure and adsorption behavior, significantly affects their dispersing and slump-retaining performance [15,36,37,38]. The comparatively favorable behavior of W7 is consistent with these mechanisms. The PCE-based system exhibited the lowest relative slump loss after 60 min among the correctly produced mixtures. This supports the interpretation that admixture type and compatibility, rather than dosage alone, are important for maintaining concrete workability [12,15,17,37]. The W3 mixture provides an important practical example of the consequences of uncontrolled water addition. Although unintended water ingress virtually eliminated the measured slump loss after 60 min, the mean 28-day compressive strength decreased to 24.7 MPa. Similar adverse effects of additional water, bleeding and changes in plastic shrinkage behavior have been reported by Yakoubi et al. [11]. The present results therefore reinforce the practical principle that workability retention should be controlled through appropriate mixture design and admixture technology rather than through uncontrolled addition of water [11,12,36]. At the same time, the results demonstrate that the w/c ratio cannot independently predict plastic shrinkage behavior. W5 had the highest w/c ratio and lowest compressive strength, yet under moderate environmental exposure W5-P1 developed only four cracks. Conversely, W4, with a lower w/c ratio, exhibited considerably more severe cracking under hot and dry exposure. Similar multi-factor interactions between w/c ratio, temperature, curing and shrinkage have been reported under field conditions [10,11,39,40].

### 4.2. Environmental Drying Potential as a Governing Factor

The W4 and W5 series provide a useful contrast between two full-scale field-production scenarios, but not a controlled environmental experiment. W4 was associated with air temperatures reaching 35.2 °C, relative humidity of approximately 28–30%, direct solar exposure and wind, whereas W5 was cast at approximately 22 °C under cloudy conditions. W4-P1 developed 19 visible cracks after 240 min and W5-P1 developed four. These observations are consistent with literature describing increased plastic-shrinkage risk under hot, dry and windy exposure [1,3,8], but the present data cannot assign the difference solely to weather because mixture composition, w/c ratio, admixture system, and reinforcement configuration also differed. The Uno-based potential evaporation rate was calculated as 0.22 kg/m^2^/h for W5 and 0.71 kg/m^2^/h for the initial W4 conditions. A 1.17 kg/m^2^/h value obtained using an approximate 40 °C surface-temperature field estimate is retained only as an illustrative sensitivity calculation because the surface-temperature measurement protocol and solar irradiance were not documented sufficiently for reproducible analysis. Mechanistic references to bleeding, settlement and capillary pressure are therefore interpreted from the literature [1,3,6,7,11,18] rather than presented as quantities measured in this experiment.

### 4.3. Case Observation: Surface Finishing in the Anomalous W3 Batch

The W3 slabs provide a case observation of two finishing procedures applied to the same anomalous concrete batch. W3-P1, finished using a vibrating screed, developed 10 visible cracks, whereas W3-P2, manually finished using a hand trowel, developed four. Because W3 experienced unintended water ingress and because *n* = 1 for each finishing condition, this difference is not statistically generalizable and cannot establish a causal effect of the finishing method. Possible explanations involving bleeding, surface-layer formation, settlement, or local restraint are mechanistic interpretations based on previous literature [3,11,18,33], as these processes were not directly measured in the present study.

### 4.4. Case Observation: Reinforcement Configuration

The W4 slabs also provide only an individual case comparison of two reinforcement configurations. The slab containing a single Ø12 mm mesh developed 19 visible cracks, whereas 13 cracks were recorded for the slab containing the standard double Ø6 mm mesh. Because both bar diameter and the overall reinforcement arrangement changed simultaneously and only one slab represented each configuration, the difference cannot be attributed to bar diameter or used to quantify a reinforcement effect. Plastic settlement and local restraint above reinforcing bars are plausible mechanisms reported in the literature [3,18,33], but they were not directly measured in this experiment.

Accordingly, the W4 reinforcement comparison is retained solely as a case-specific field observation. It indicates that different reinforcement arrangements can coexist with different visible crack counts under otherwise similar W4 production conditions, but it does not demonstrate a universal quantitative relationship between reinforcement geometry and plastic shrinkage cracking.

### 4.5. Curing Observations Under the Investigated Field Conditions

Among the investigated curing scenarios, PE sheeting showed the most consistent final visible-crack outcome: all nine PE-protected slabs were free of visible cracks at final inspection. Water-cured slabs also showed low final crack counts (zero or one), while the membrane-forming compound produced more variable outcomes. These observations are consistent with the physical principle that limiting surface moisture loss can reduce plastic-shrinkage risk [9,20,21,39]. However, the curing methods were not compared in a balanced factorial design, group sizes were unequal, and monitoring protocols differed for PE-covered and some compound-treated slabs. The results therefore do not support a statistical ranking of curing methods; PE sheeting is described only as the most consistent method under the investigated conditions.

### 4.6. Combined Field-Production Scenarios

The combined results show that material, environmental, construction, and curing conditions occurred simultaneously in the full-scale production program. The W4–W5 comparison illustrates this confounding clearly: W5 had w/c = 0.85 and a 28-day cube compressive strength of 24.6 MPa but developed four visible cracks under its moderate field scenario, whereas W4 had a lower w/c ratio and W4-P1 developed 19 cracks during a much hotter and drier scenario. This does not demonstrate that one factor dominated another, nor that high w/c protects against plastic shrinkage. It shows only that visible crack occurrence cannot be interpreted from w/c or mechanical strength in isolation when exposure and construction conditions also differ. Literature on bleeding, evaporation, settlement and capillary pressure provides plausible mechanisms for such observations [1,3,6,10,11,18,39], but these mechanisms were not directly measured here. The scientific value of the present study is therefore the documentation of full-scale field-production behavior rather than quantitative determination of factor interactions.

### 4.7. Practical Implications for Ready-Mixed Concrete Construction

The experimental findings support several practical considerations for ready-mixed concrete construction. Workability retention should preferentially be achieved through appropriate admixture technology rather than uncontrolled additional water [12,15,36,37,38]. Plastic-shrinkage risk assessment should consider the documented environmental variables—air/concrete temperature, relative humidity and wind velocity—together with surface protection [1,8,21]. The W4 observations also suggest that direct solar exposure may increase drying potential, but the present study did not document solar irradiance or a reproducible surface-temperature measurement protocol and therefore does not quantify this effect. Early surface protection should be planned as part of the concreting procedure; within the investigated scenarios, PE sheeting was associated with the most consistent crack-free final observations, without implying a statistically proven superiority over all alternative curing methods.

Finally, workability, compressive strength and resistance to plastic shrinkage should be regarded as distinct performance characteristics. A mixture may exhibit excellent workability retention but inadequate mechanical performance if additional water has been introduced, while concrete with relatively poor compressive strength may exhibit limited plastic cracking when environmental drying is low.

### 4.8. Limitations and Research Needs

The full-scale character of the study increases its practical relevance but limits strict experimental control. Environmental variables differed between casting days, and several comparisons involved simultaneous changes in material, exposure, and construction conditions. The study should therefore be interpreted as a series of comparative field-production cases rather than a factorial experiment capable of separating independent effects or statistically quantifying interactions. Only one slab represented each surface-finishing, reinforcement, and water-curing timing condition; these comparisons are case-specific observations. W3 was affected by unintended water ingress and is retained only as an unplanned field-production event, not as a controlled admixture or finishing experiment. PE-covered slabs were not repeatedly uncovered during the first 240 min, and W6-P5–W6-P10 were subjected only to final crack inventory; consequently, final crack count and crack-development kinetics are not methodologically equivalent and are not interpreted as such. Visible crack count also does not capture crack width, total crack length, cracked area, or spatial distribution. Future studies should use replicated full-scale designs with independent factor control where feasible and should supplement visual observations with optical microscopy or digital image processing. The present environmental record provides series-level ranges but not a fully time-resolved casting chronology for every slab, and the approximate solar-heated surface-temperature estimate lacks sufficiently documented instrumentation and irradiance data. These limitations should be addressed in future field campaigns.

## 5. Conclusions

The present study documented plastic shrinkage cracking and 28-day cube compressive strength for seven concrete mixtures designed for strength class C30/37 and 28 full-scale slabs produced under realistic ready-mixed concrete plant conditions. Because mixture composition, environmental exposure, finishing, reinforcement, and curing were not varied independently in a balanced factorial design, the conclusions are formulated as field-scenario associations rather than cause-and-effect relationships:

1. Reducing or eliminating water-reducing admixtures substantially increased the water demand required to maintain the target workability. The actual w/c ratio increased from 0.58 for the reference mixture W1 to 0.85 for the admixture-free W5 mixture. This demonstrates that maintaining a comparable initial slump through additional water is not technologically equivalent to maintaining it through an effective admixture system.

2. The increase in w/c ratio resulted in a pronounced reduction in compressive strength. The highest 28-day mean compressive strength, 41.4 MPa, was obtained for W1 at w/c = 0.58, whereas W5 reached only 24.6 MPa at w/c = 0.85, corresponding to a reduction of approximately 41%. The W3 technological anomaly, involving unintended water ingress, similarly resulted in a low mean strength of 24.7 MPa despite excellent apparent slump retention.

3. Initial workability alone was not sufficient to characterize concrete technological performance. Mixtures with comparable initial slump values showed markedly different slump retention after 60 min. The PCE-based W7 mixture exhibited the lowest relative slump loss among the correctly produced mixtures, confirming the importance of an appropriate admixture system for maintaining workability without increasing water content.

4. Visible plastic shrinkage cracking could not be interpreted from w/c ratio alone within the investigated field-production scenarios. W5, despite having the highest w/c ratio and lowest mean cube compressive strength, developed fewer visible cracks under its moderate scenario than W4-P1 under the severe W4 scenario. Because the mixtures and reinforcement also differed, this observation does not isolate an independent environmental or mixture effect.

5. The severe W4 field-production scenario was associated with substantially greater visible cracking than the more moderate W5 scenario: W4-P1 developed 19 visible cracks and W5-P1 developed 4. The calculated potential evaporation rates for the documented initial conditions were 0.71 and 0.22 kg/m^2^/h, respectively. This association should not be interpreted as proof that environmental drying was the dominant independent factor because material and construction variables were confounded.

6. The finishing and reinforcement comparisons are case-specific observations only. In the anomalous W3 batch, the vibrating-screed slab had 10 visible cracks and the hand-trowelled slab had 4; in W4, the single-Ø12-mm-mesh slab had 19 and the standard double-Ø6-mm-mesh slab had 13. With *n* = 1 per configuration, and with W3 affected by unintended water ingress, these comparisons do not support statistical or causal conclusions regarding finishing technique or reinforcement diameter.

7. PE sheeting was the most consistent surface-protection method under the investigated conditions: all nine PE-protected slabs were free of visible cracks at final inspection. The two water-cured slabs had zero or one visible crack, whereas the membrane-forming compound showed more variable final counts. Because group sizes and monitoring protocols differed, these observations do not constitute a statistical ranking of curing methods.

8. For full-scale concrete construction, effective control of plastic shrinkage requires an integrated technological approach. Workability should be maintained through suitable admixture technology rather than uncontrolled water addition, environmental drying conditions should be monitored using parameters including concrete surface temperature, relative humidity and wind velocity, and curing should be implemented as early and continuously as practicable.

Overall, the study provides full-scale evidence that visible plastic shrinkage cracking under field-production conditions reflects the complete production and exposure scenario. The results support careful control of mixture water demand, environmental drying potential, construction practice, and early surface protection, while also showing that the present non-factorial dataset should not be used to assign independent causal weights to these variables.

## Figures and Tables

**Table 1:** Actual composition of the concrete mixtures.

Component [kg/m^3^]	W1	W2	W3	W4	W5	W6	W7
Cement	271.23	269.33	274.67	268.67	266.00	286.00	270.33
Fly ash	36.00	38.00	40.00	40.00	34.67	39.20	39.17
Sand 0–2 [mm]	709.76	702.08	722.56	721.03	711.68	709.12	710.76
Aggregate 2–8 [mm]	418.44	623.70	609.18	615.12	632.94	621.98	620.06
Aggregate 8–16 [mm]	533.28	521.40	535.92	524.04	520.08	530.51	529.27
CHRYSO Plast 229	1.40	2.70	1.28	1.37	-	2.86	-
CHRYSO Delta 131	2.70	-	-	-	-	-	-
MC-TechniFlow25	-	-	-	-	-	-	2.70
Total water	157.19	192.25	205.67	209.81	225.43	181.19	175.91
w/c	0.58	0.71	0.75	0.78	0.85	0.63	0.65

**Table 2:** Full-scale slab experimental program.

Mixture	Number of Slabs	Main Experimental Condition
W1	2	Reference mixture, curing compound vs. no curing
W2	2	No superplasticizer, no surface protection
W3	2	Reduced plasticizer dosage, unintended additional water, vibrating screed vs. hand trowel
W4	2	Reduced plasticizer dosage, different reinforcement configurations under severe summer exposure
W5	2	No chemical water-reducing admixtures, no surface protection
W6	10	Comparative assessment of water curing, PE sheeting and membrane-forming curing compound
W7	8	Alternative PCE superplasticizer, PE-sheet curing
Total	28	

**Table 3:** Environmental conditions during production of the individual concrete variants.

Variant	Air Temperature [°C]	RH [%]	Concrete Mixture Temperature [°C]	Wind Velocity [m/s]
W1	22.9–24.1	63–71	24.1–26.2	1.6–1.8
W2	21.2–21.9	49–52	22.4–23.9	3.9–5.1
W3	23.2–24.8	33 (single recorded value)	24.7–26.2	3.4–3.9
W4	33.4–35.2	28–30	21.1–32.1	1.6–2.2
W5	21.9–22.1	50–53	22.9–23.2	1.1–1.4
W6	25.2–26.6	52–55	26.5–27.9	2.0–2.8
W7	19.8–20.4	64–69	22.0–22.2	0.3–0.7

**Table 4:** Slump retention of the investigated concrete mixtures.

Variant	Initial Slump, 0 min [mm]	Slump After 60 min [mm]	Slump Loss [mm]	Relative Slump Loss [%]
W1	155	125	30	19.3
W2	155	120	35	22.6
W3	150	150	0	0.0
W4	155	110	45	29.0
W5	160	100	60	37.5
W6	140	110	30	21.4
W7	160	135	25	15.6

**Table 5:** Compressive strength of the investigated concrete mixtures after 28 days.

Variant	Actual w/c	Mean Compressive Strength [MPa]	SD [MPa]
W1	0.58	41.4	0.87
W2	0.71	33.4	1.88
W3 *	0.75	24.7	0.66
W4	0.78	30.8	0.95
W5	0.85	24.6	0.93
W6	0.63	38.3	1.29
W7	0.65	36.8	0.72

* W3 was affected by unintended water ingress during concrete production.

**Table 6:** Development of plastic shrinkage cracking for different surface-finishing techniques.

Time After Casting [min]	W3-P1 Vibrating Screed	W3-P2 Hand Trowel
0	0	0
30	0	0
60	0	0
90	0	0
120	5	2
150	6	3
180	9	4
210	10	4
240	10	4

**Table 7:** Development of plastic shrinkage cracking for different reinforcement configurations.

Time After Casting [min]	W4-P1 Ø12 mm	W4-P2 Ø6 mm
0	0	0
30	0	0
60	0	0
90	5	4
120	8	7
150	11	10
180	14	12
210	16	13
240	19	13

**Table 8:** Development of plastic shrinkage cracking under severe and moderate environmental conditions.

Time After Casting [min]	W4-P1 Severe Exposure	W4-P2 Moderate Exposure
0	0	0
30	0	0
60	0	0
90	5	2
120	8	3
150	11	4
180	14	4
210	16	4
240	19	4

## Data Availability

The original contributions presented in this study are included in the article. Further inquiries can be directed to the corresponding author.
